# Visible light photocatalytic reduction of aldehydes by Rh(iii)–H: a detailed mechanistic study†
†Electronic supplementary information (ESI) available: Detailed experimental procedures of the photocatalytic reactions, additional spectral data, calculations, photochemical measurements and mechanistic studies and schemes. See DOI: 10.1039/c4sc03709j



**DOI:** 10.1039/c4sc03709j

**Published:** 2015-01-06

**Authors:** T. Ghosh, T. Slanina, B. König

**Affiliations:** a Institute of Organic Chemistry , University of Regensburg , D-93040 Regensburg , Germany . Email: Burkhard.Koenig@ur.de; b Department of Chemistry , Faculty of Science , Masaryk University , Kamenice 5 , 62500 Brno , Czech Republic; c Research Centre for Toxic Compounds in the Environment , Faculty of Science , Masaryk University , Kamenice 5 , 62500 Brno , Czech Republic

## Abstract

The chemoselective photoreduction of aldehydes in the presence of ketones was achieved using triethanolamine (TEOA) as sacrificial electron donor, proflavine (PF) as photocatalyst and [Cp*Rh(iii)(bpy)Cl]Cl (Rh_cat_) as mediator. The reducing agent, which reacts with the carbonyl group was found to be [Cp*Rh(iii)(bpy)H]Cl (Rh(iii)–H). Contrary to formate-based reduction, its slow photochemical *in situ* generation enables to kinetically distinguish aldehydes from ketones. The inherent reactivity difference of the carbonyl compounds is transferred by the method into synthetically useful reaction selectivities. The substrate scope is broad with excellent yields. A detailed study of the reaction mechanism reveals that the photoreduction of the PF triplet and the subsequent reduction of the Rh_cat_ leading to Rh(iii)–H represents the major reaction pathway, which is highly oxygen sensitive. The oxidative quenching of the PF singlet state by Rh_cat_ is a competing mechanism, which prevails in non-degassed systems.

## Abstract 2

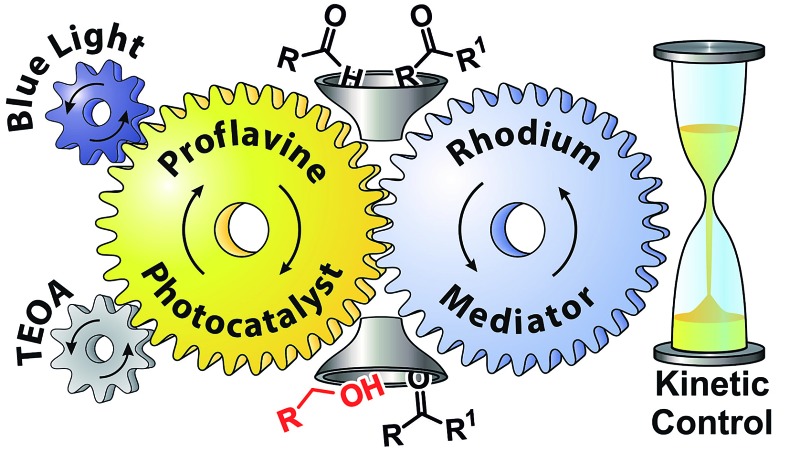
The slow visible light mediated generation of a rhodium hydride allows the chemoselective reduction of aldehydes in the presence of ketones. Electron transfer from the chromophore to the metal complex proceeds *via* a radical anion intermediate or a solvated electron as two competing reaction pathways.

## Introduction

Aldehydes and ketones are similar in reactivity. The development of methods for the chemoselective reduction of aldehydes in the presence of ketones has therefore received considerable attention.^1,2^ Employing NaBH_4_ as reduction reagent, selectivity can be achieved only at very low temperatures (–78 °C)^3,4^ or by using additives such as thiols,^5^ metal salts,^6^ resins,^7^ PEG^8^ or Na_2_CO_3_ in water.^9^ Various modified borohydrides are known to allow chemoselective reduction of aldehydes in the presence of ketones. For example, tetraalkylammonium borohydride can reduce aldehydes in the presence of ketones to its corresponding alcohol, but with only low selectivity.^10^ Na(AcO)_3_BH^11^ and *n*-Bu_4_N(AcO)_3_BH^12^ were used to reduce aldehydes in the presence of ketones with a high selectivity, but rather harsh reduction conditions, such as reflux in benzene, are required. In recent past, chemists started to modify borohydrides^13^ with sterically hindered substituents and different electron-withdrawing groups, which are then able to distinguish between the carbonyl groups of aldehydes and ketones. Most of these modified borohydrides require special reagents and methods to prepare. Moreover, in all these hydride reductions the reducing agent was used stoichiometrically. In 2006 Casey *et al.* introduced the catalytic chemoselective hydrogenation of aldehydes over ketones in non-polar solvent at elevated temperature, which was demonstrated with only one example: the reduction of benzaldehyde in the presence of acetophenone.^14^ In 2012 McCulla *et al.* reported^15^ photo-chemical chemoselectivity of aryl aldehydes in the presence of alkyl aldehydes and aryl ketones. They used a polymeric heterogeneous photocatalyst with a tail absorption (400–440 nm) in the visible part of the spectrum. However, by this method they were able to achieve only low conversion of starting materials with low overall yields of the corresponding alcohols for both neutral and electron rich aldehydes. Moreover, they often observed the benzoin condensation as a side reaction.

**Fig. 1:**
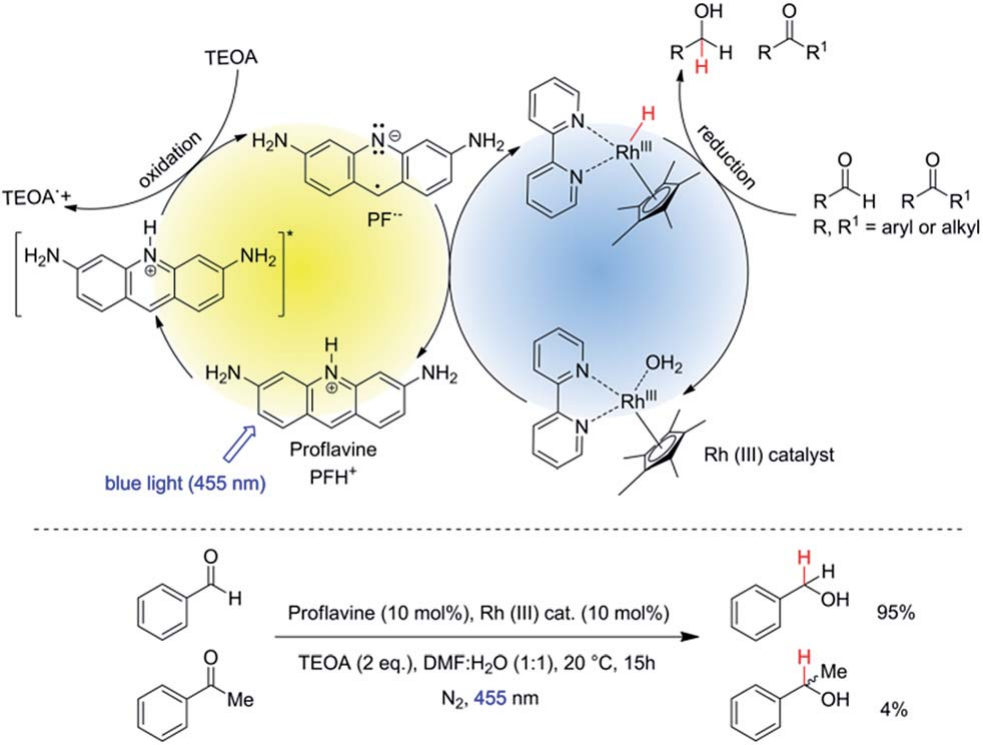
Top: schematic representation of the photocatalytic cycle with mediator cycle involving PF as photocatalyst and [Cp*Rh(iii)(bpy)Cl]Cl as mediator. Bottom: high chemo-selectivity for benzaldehyde in the presence of acetophenone.

Herein, we report the chemoselective visible light induced photocatalytic hydride reduction of aldehydes in the presence of ketones. Our photocatalytic system offers, in comparison to previously published methods, a robust selectivity, which can differentiate aldehydes from ketones over a broad reactivity range. Park and Nam have introduced^16^ a photocatalytic system using PF (3,6-diaminoacridine) as photocatalyst and Rh_cat_ as a mediator for the regeneration of NADH from NAD^+^ produced by enzymatic synthesis of l-glutamate demonstrating an artificial photosynthetic approach. We modified the system for synthetic purposes. The schematic mechanism is shown in Fig. 1, upper part. PF is a well-known acridine dye studied in detail for its ability to bind with DNA.^17^ It has also been used as a promising molecule for the photogeneration of hydrogen.^18^ Rh_cat_ has been first described by Youinou and Ziessel in 1989.^19^ Since then it has frequently been used as a hydride transferring agent for cofactor regeneration.^20^ Unlike other hydrides, it exhibits an outstanding regioselectivity in the reduction of NAD^+^.^21^ It has also been used for the chemical reduction of both aldehydes and ketones by hydride transfer from formic acid.^22,23^ We photochemically generate the same hydride reducing agent, Rh(iii)–H as in the formate-based reduction. However, due to its slow *in situ* generation, we maintain a low concentration of Rh(iii)–H in the reaction medium, which then kinetically distinguishes between aldehydes and ketones with a high selectivity (Fig. 1, bottom).

## Results and discussion

### Synthetic investigations

The reaction conditions were optimized using benzaldehyde as a substrate. The selected results are summarized in the Table 1. The yields were determined by GC/FID after 15 hours of irradiation at 455 nm. The reactions in anhydrous organic solvent (Table 1, entries 1 and 2) did not yield a significant amount of product as water is required as a proton source for generation of Rh(iii)–H.^24^ Both aqueous acetonitrile and DMF gave good yields and DMF/H_2_O (1 : 1, v/v) was chosen for further studies as the aliphatic substrates dissolve better in the reaction medium. The yields of benzyl alcohol were highest in case of 10 mol% of both PF and Rh_cat_ (Table 1, entry 5). Using 5 mol% of both PF and Rh_cat_ we obtained a similar result for the benzaldehyde reduction (Table 1, entry 9), but we increased the catalysts loading to 10 mol% to accelerate the reduction rate of aliphatic substrates.

**Table 1:** Optimization of reaction conditions

Entry	Proflavine (mol%)	Rh_cat_ (mol%)	TEOA (eq.)	Solvent	Yield after 15^*a*^ h (%)
1	10	10	2	Dry MeCN	<1
2	10	10	2	Dry DMF	7
3	10	10	2	DMF/H_2_O (1 : 2)	83
4	10	10	2	DMF/H_2_O (2 : 1)	61
**5**	**10**	**10**	**2**	**DMF/H**_**2**_ **O (1** : **1)**	**97**
6	10	10	2	MeCN/H_2_O (1 : 1)	80
7	5	10	2	DMF/H_2_O (1 : 1)	86
8	10	5	2	DMF/H_2_O (1 : 1)	73
9	5	5	2	DMF/H_2_O (1 : 1)	95
10	10	10	1	DMF/H_2_O (1 : 1)	35
11	10	10	3	DMF/H_2_O (1 : 1)	81

^*a*^GC/FID determined yield with appropriate internal standard.

To investigate the role of each component of the photocatalytic system we performed a series of control experiments. The results are summarized in Table S1.† The data clearly show that each component is essential for the reaction progress. The reaction without degassing (Table S1,† entry 6) yields about 30% of the product. This has been further studied and will be discussed in the mechanistic part. Reactions in hydrogen atmosphere did not yield any product (Table S1,† entries 7 and 8) from which it is evident that no direct hydrogenation occurs.

Various aromatic and aliphatic aldehydes and ketones were tested as substrates in our catalytic system (Table 2). For all substrates the optimized reaction conditions were used (Table 2, entry 5). The reaction rate could be accelerated by a factor of 5, without affecting the selectivity (Table 2, entries 1–3) using a flow reactor, which delivers the incident light more efficiently to the whole volume of the reaction mixture. Excellent yields were obtained for neutral, electron rich and electron poor aldehydes, whereas the corresponding ketones remained unreacted. Using an activated ketone as one reactant, we performed the reduction reactions varying the other reactant from electron-poor to electron-neutral to electron-rich aldehyde with notable selectivity (Table 2, entry 8–10). The selectivity was observed not only for a mixture of aldehyde and ketone, but also for a bifunctional molecule (Table 2, entry 11). Somewhat lower yield in entry 11 is caused by a side reaction leading to a pinacol-type product (detected by HPLC-MS, see Fig. S44†). In entry 12 a lower yield was obtained, because of decomposition of the substrate, which is not related to the photoreaction.

**Table 2:** Substrate scope

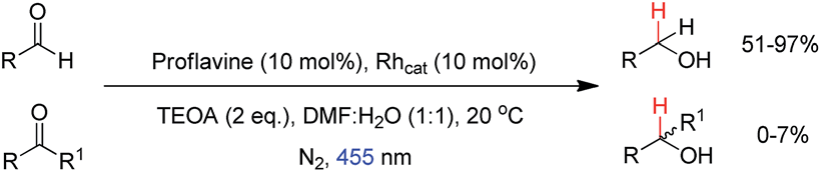				
Entry	Substrate	Reaction type	Time (h)	Yield
1	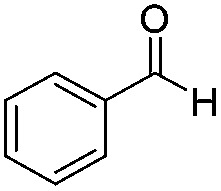	Batch	15	97
		Flow	3.5	91
2	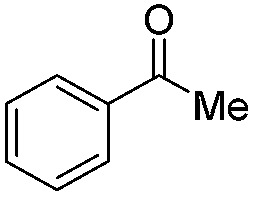	Batch	15	7
		Flow	3.5	4
3	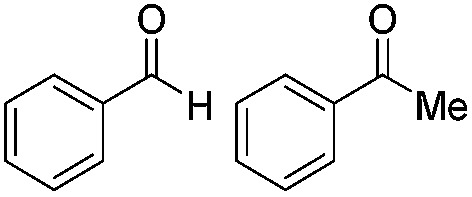	Batch	15	95 (4)^*a*^
		Flow	3.5	82 (<1)^*a*^
4	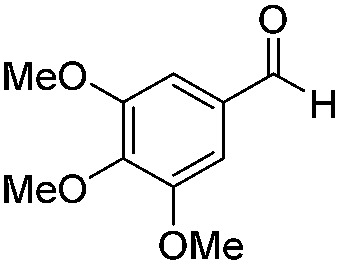	Batch	25	95
5	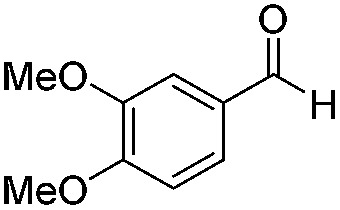	Batch	25	83
6	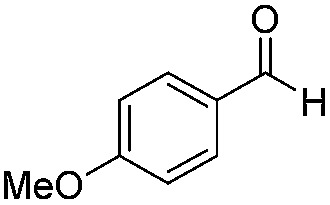	Batch	32	84
7	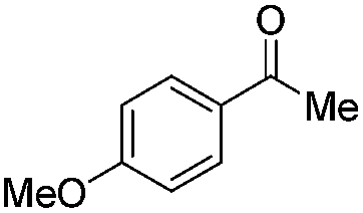	Batch	32	3
8	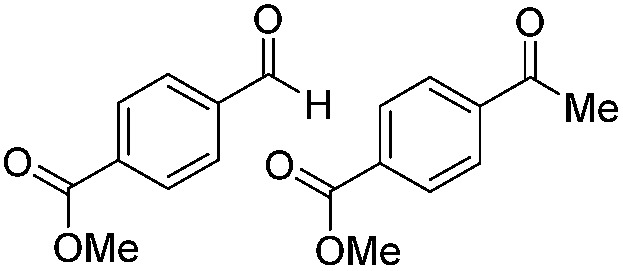	Batch	18	76 (2)^*a*^
9	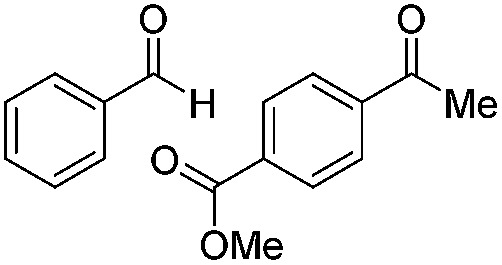	Batch	16	91 (2)^*a*^
10	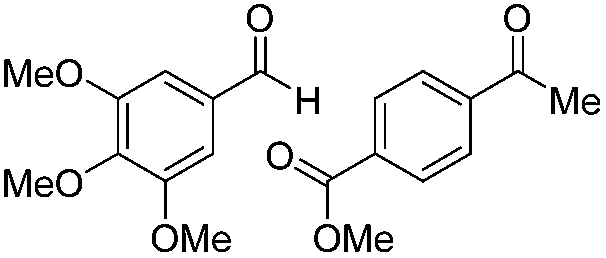	Batch	25	93 (4)^*a*^
11	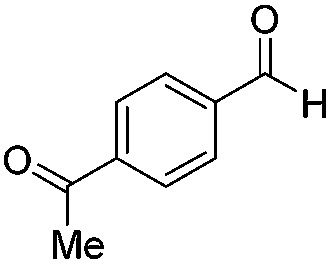	Batch	16	51 (<1)^*b*^
12	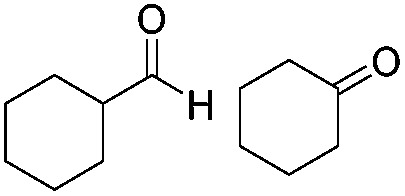	Batch	42	56 (3)^*a*^

^*a*^Yields of ketone reductions.

^*b*^Yield of doubly reduced product.

The rate of reduction is partially dependent on the electron density of the aldehyde functionality. That indicates that the hydride transfer from Rh(iii)–H to the carbonyl compound is the rate-determining step. The correlation of reaction yields, reduction potentials and Hammett's sigma values is shown in Fig. S8.† Generally, the photoreduction is slower for electron-rich aldehydes, but no clear trend was observed. Ketones are almost unreacted, which is mainly caused by steric effects. Rh_cat_ is sufficiently crowded to create selectivity even between similar substrates, which was demonstrated on various NAD^+^ model compounds.^21^

The catalytic system also reduces imines (see Table S3†). Dry DMSO was found to be the most suitable solvent and the addition of thiourea (1 eq.) accelerated the reaction significantly by hydrogen bond activation of the imine.^25^

The reaction selectivity was compared with known systems. Rh_cat_ has been recently used for chemical reductions of both aldehydes and ketones.^22,23^ The selectivity is reported only marginally.^22^ The reactions were accomplished in biphasic conditions without any phase transfer catalyst. The reduction was fast even with low catalyst loadings (∼0.5 mol%). We therefore examined the selectivity of Rh(iii)–H generated chemically using formate aqueous buffer as a hydride source. The results are shown in Table S2.† After 15 minutes the benzaldehyde is efficiently reduced, whereas the conversion of acetophenone is only 32%. Contrary to the formate-based system our photocatalytic reduction is slower and the reaction can be easily stopped after the aldehyde is reduced and the ketone is almost intact. The aldehyde–ketone selectivity depends on the reaction conversion and therefore the ratio of reduction products is influenced by the reaction time. The kinetics of the reaction is described in more detail in the ESI (Scheme S3 and Fig. S12†).

### Mechanistic investigations

The photophysical properties of PF have been studied in detail. In solution the dye is prone to dimerization (*K*_D_ = 500 M^–1^) and its molar absorptivity is concentration dependent from *c* ∼ 10^–4^ M.^26^ At physiological pH, PF is protonated at the central nitrogen atom N-10; PFH^+^ (p*K*_a_ = 9.5).^27^ PFH^+^ absorbs at 443 nm and has a strong fluorescence (*Φ*_fl_ = 0.39, *λ*_em_ = 508 nm)^28^ whereas the neutral form (PF) absorbs at 393 nm and exhibits no fluorescence§
§PF is weakly fluorescent till pH = 11.5 which corresponds to the p*K*_a_ of the singlet excited state. K. Kalyanasundaram; D. Dung, *J. Phys. Chem.*, 1980, **84**, 2551. (see Fig. S13 and S16†). PFH^+^ has interesting emission properties. It exhibits strong prompt fluorescence from the singlet state, ^1^[PFH^+^]* (Fig. S45†), thermally dependent delayed E-type fluorescence (fl^E^) originating from thermal repopulation of ^1^[PFH^+^]* from ^3^[PFH^+^]*, concentration dependent delayed P-type fluorescence (fl^P^) caused by triplet–triplet annihilation with energy transfer,¶
¶^3^[PFH^+^]* + ^3^[PFH^+^]* → ^1^[PFH^+^]* + ^1^[PFH^+^]. and light intensity dependent photoionization recombination delayed fluorescence (fl^PIR^) which occurs after recombination of ion pair [PFH˙]^2+^···*e* ^–^(solv.) created by photoionization from ^1^[PFH^+^]*.^29^ Phosphorescence from the triplet state is the most significant emission with maximum intensity at 570 nm till 197 K and is negligible above 253 K.^29^

Photoinduced electron transfer (PET) occurs between ^3^[PFH^+^]* and an appropriate electron donor. The redox potential of ^3^[PFH^+^]* can be estimated using the Rehm–Weller equation from the measured ground state redox potential (*E*_0_ = –0.74 V *vs.* SCE, Fig. S6†) and its triplet energy (*λ*_phosph._ = 570 nm, ∼2.17 eV) resulting in +1.44 V *vs.* SCE.‖
‖This value corresponds well with the published potential (+1.36 V). M. P. Pileni; M. Grätzel, *J. Phys. Chem.*, 1980, **84**, 2402. Electron-rich compounds like amines can serve as electron donors for PET. TEOA (*E*_0_ = +0.76 V *vs.* SCE**
**E_0_ = +0.80 V *vs.* Ag/AgCl.)^30^ is easily†
†Δ*G*° = –*e* × (–0.76 V + 1.44 V) – 0.08 eV = –0.76 eV ∼ –73.3 kJ mol^–1^, according to *J. Am. Chem. Soc.*, 1999, **121**, 1681–1687. oxidized by ^3^[PFH^+^]* creating TEOA˙^+^ and a reduced proflavine radical (PFH˙). The back electron transfer does not occur due to the fast deprotonation of TEOA˙^+^.^31^

**Fig. 2:**
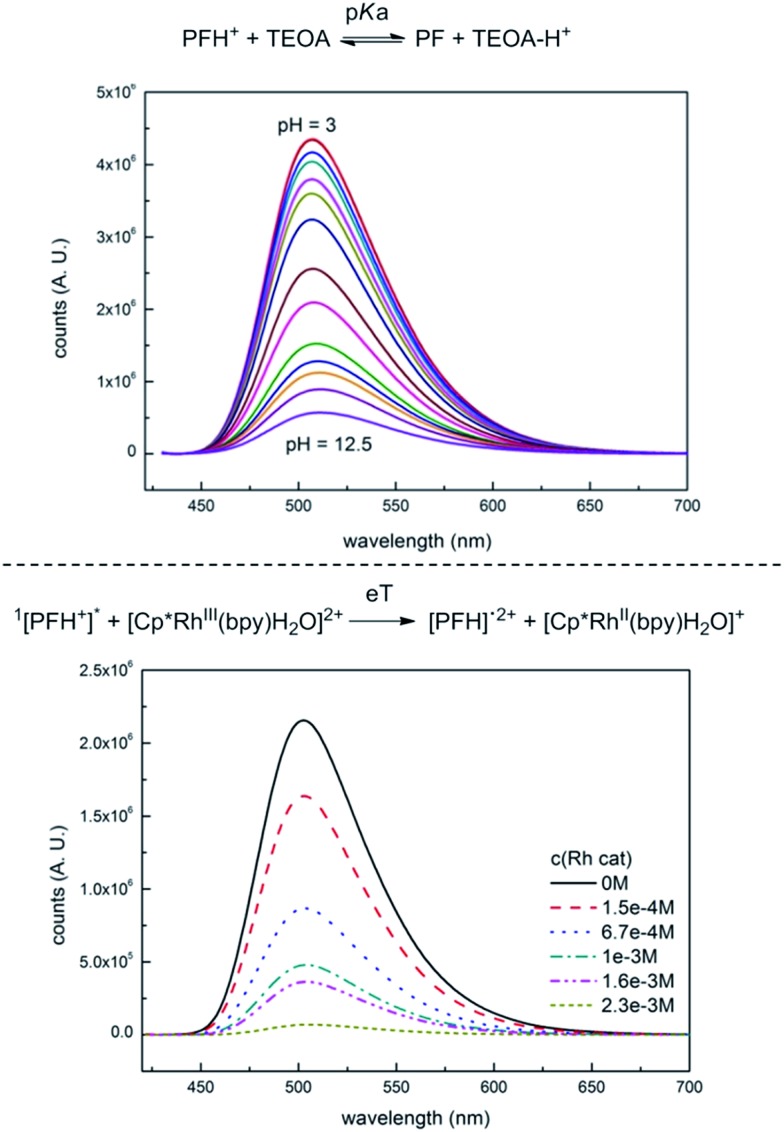
Fluorescence quenching of PF with TEOA (upper part) and Rh_cat_ (lower part).

Interaction of PF with TEOA in aqueous media has been studied by measuring its fluorescence. Titration of PF solution (aq., *c* = 5.0 × 10^–6^ M) with TEOA or TEA resulted in a decrease in fluorescence intensity as observed by Basu *et al.* ^32^ This would indicate that TEOA is quenching the singlet excited state by PET and would be in contradiction with the well-established PET from PF triplet.^31^ The UV spectra (Fig. S15†) indicate the formation of a new species with an absorption peak at 393 nm, which corresponds to PF formed by a simple acidobasic equilibrium, which is also supported by the UV pH titration (Fig. S13†) and fluorescence pH titration (Fig. 2, upper part). The distribution of the respective acidobasic forms calculated from both pH and TEOA titration corresponds to each other (Fig. S20†). We did not observe the formation of PFH^+^···TEA ground-state complex as proposed by Basu *et al.* ^32^

Fluorescence quenching of PF with Rh_cat_ is shown in Fig. 2, lower part. Unlike TEOA, the Rh catalyst does not interact with PF in the ground state. Fluorescence was quenched at relatively high concentrations.‡
‡300 eq. of Rh catalyst *vs.* PFH^+^, Stern–Volmer quenching constant is (2260 ± 30) M^–1^. The quenching constant was dependent on the excitation beam intensity. This indicates that Rh_cat_ is quenched by photoionized electrons, which are originally responsible for the photoionization recombination delayed fluorescence (fl^PIR^). The contribution of the photoionization recombination delayed fluorescence to the overall emission was determined by measuring the dependence of the relative fluorescence yield on the intensity of excitation light. The light intensity was kept below the saturation limit so that all of the excitation light was absorbed by the sample. Increasing intensity of the excitation light leads to a non-linear increase of the fluorescence intensity, which corresponds to the fl^PIR^ (Fig. S22†).

**Fig. 3:**
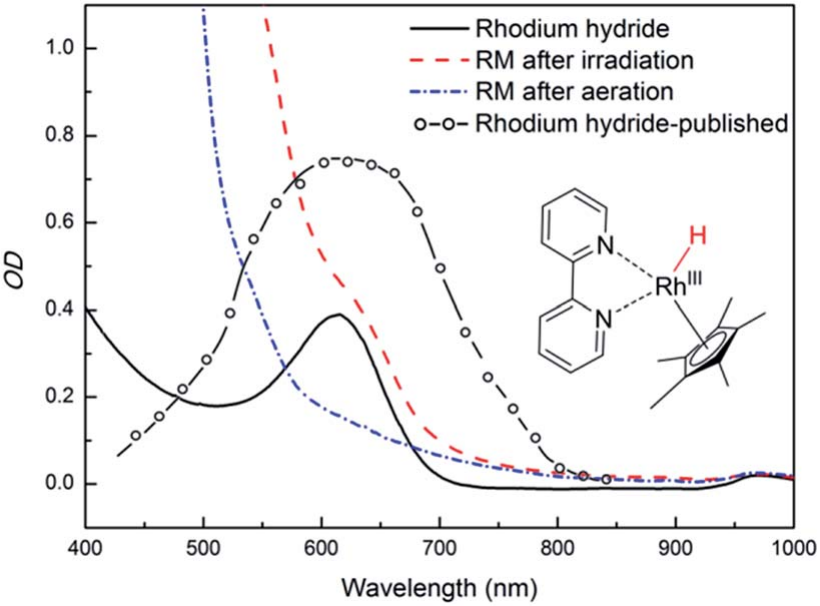
Spectroscopic evidence of presence of Rh(iii)–H in the photocatalytic system (left side). Spectra of a typical reaction mixture after irradiation (dashed red line), after bubbling with air (dash – dotted blue line), of a prepared Rh(iii)–H standard (solid black line), and a published^24^ spectrum (open circles + dashed line; redrawn from the original) are shown.

The properties of rhodium mediator were studied in detail. Rh_cat_ is a water-soluble air-stable d^6^ metal complex, which undergoes a ligand exchange after its dissolution in water. The catalytic active form [Cp*Rh(iii)(bpy)(H_2_O)]Cl_2_ has its maximum absorption at 355 nm and a tail absorption in the visible region (*λ*_tail_ ∼ 420 nm, Fig. S25†). Its absorption in the blue region (*λ* = 455 nm) is weak§
§Measured molar absorptivities are: *ε* ^455^(PFH^+^) = 28 600; *ε* ^455^(Rh^III^cat) = 120. and it does not interfere with PFH^+^. The reducing species has been described as a metal hydride complex, Rh(iii)–H. It was confirmed as a key intermediate in the formate-based reductive catalytic system generated by direct hydride transfer from HCOO^–^.^33^ It has also been proposed as a reducing agent in photocatalytic systems coupled with various dyes.^16,34,35^ To identify Rh(iii)–H in our reduction system we prepared Rh(iii)–H independently from the reaction with formate ions. After dissolution of Rh_cat_ in formate buffer (2 M; pH = 3.5) bubbles of CO_2_ and H_2_ were generated vigorously. The yellow solution turned blue and could be slowly re-oxidized back by O_2_. A new absorption peak at 612 nm is observed (Fig. S25†) corresponding to the previously published spectra of Rh(iii)–H (Fig. 3). Due to the vigorous gas evolution we were not able to measure the NMR spectrum for structural characterization. EPR analysis showed that the hydride complex is diamagnetic, which corresponds to the previous findings. In the UV-vis spectrum of the typical reaction mixture without substrate (Fig. 1) irradiated for 15 hours with 455 nm LED the shoulder at 612 nm corresponding to the Rh(iii)–H was observed. After purging with air the peak vanished and the spectrum changed to the initial state before irradiation (Fig. 3). This is a clear evidence for the presence of Rh(iii)–H in the reaction mixture.

Rh(iii)–H is known to produce dihydrogen upon its protonation by the solvent.^24^ We therefore examined if the hydrogen is produced in the catalytic system. We measured the composition of the gas phase above the typical reaction mixture after 15 hours of irradiation by head-space GC. Dihydrogen was present together with nitrogen used for degassing (Fig. S9†). We also examined if the presence of H_2_ in the reaction mixture could be responsible for the course of the reaction. The typical reaction mixture without TEOA was purged with oxygen-free H_2_ (Table S1,† entries 7 and 8) and was irradiated for 15 h. No product formation was observed, which indicates that the decomposition of Rh(iii)–H is an irreversible process and that carbonyls cannot be reduced by dihydrogen itself in the presence of the Rh catalyst.

To have a better insight into the mechanism we measured the kinetics of the evolution of H_2_ using benzaldehyde or acetophenone as a substrate (benzaldehyde is being reduced by Rh(iii)–H efficiently whereas acetophenone is not). The result is shown in Fig. S10.† In the photo-reduction of benzaldehyde the amount of H_2_ produced is lower (approx. by the factor of 2) than when acetophenone is used. In the first case a fraction of Rh(iii)–H (*ca.* 50%) is consumed for the reduction and the rest is decomposed by protonation.¶
¶These side reactions have similar rate constants. In the case of the ketone, where no reduction was observed, the Rh(iii)–H is solely decomposed to dihydrogen.‖
‖The ketone reduction does not efficiently compete with the decomposition. This behavior corresponds to the side reaction kinetics shown in the Fig. S12.†

**Fig. 4:**
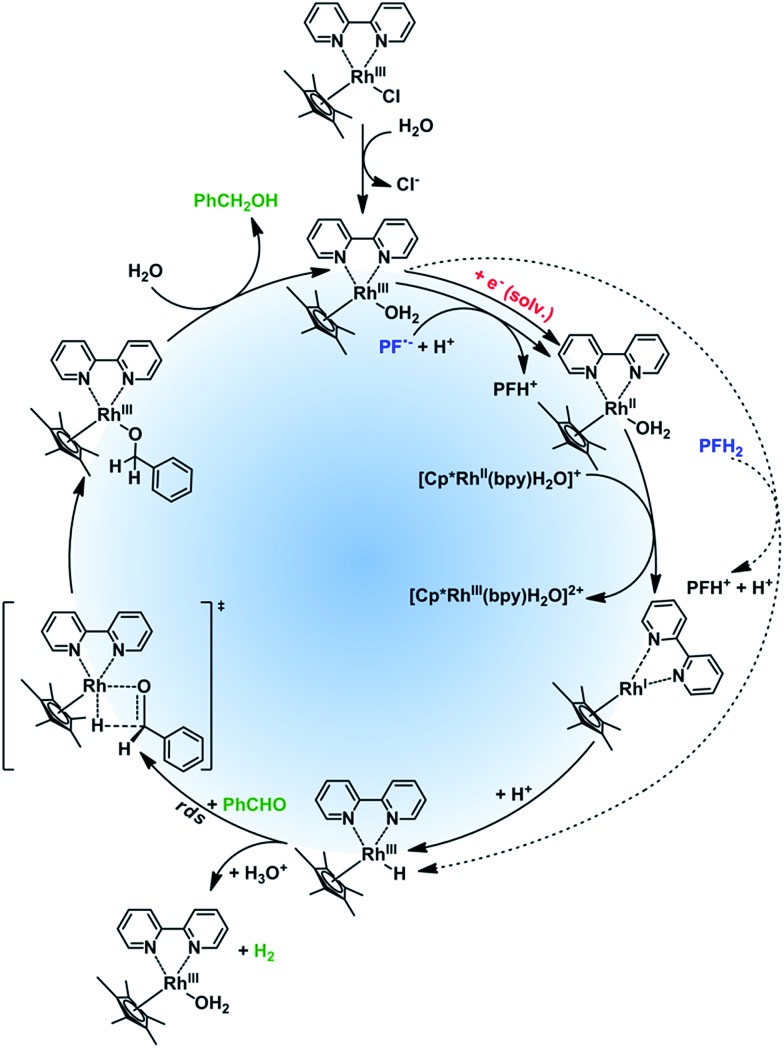
Proposed rhodium catalytic cycle, rds = rate determining step.

Based on the literature data and our experimental results we suggest the mechanism of the rhodium catalytic cycle depicted in the Fig. 4. The aqueous solution of Rh_cat_ contains [Cp*Rh^III^(bpy)H_2_O]Cl_2_, formed after the ligand exchange of Cl^–^ to H_2_O. This process is important for the catalytic activity making the central metal ion more accessible.^36^ In the next step, the rhodium aqua-complex is reduced. In principle, two different mechanisms are possible: the one electron reduction^24^ or a hydride transfer from a suitable hydrogen donor (*e.g*. HCOO^–^)^36^ have both been described in detail. The first mechanism applies for PF˙^–^ generated by PET from ^3^[PFH^+^]* and TEOA and subsequent deprotonation**
**p*K*_a_(PFH˙) = 4.5; *J. Chem. Soc., Chem. Comm.*, 1979, 1137–1138. (for p*K*_a_ values of PF species in various oxidation and excitation states see Fig. S45†). The deprotonation of PFH˙ to PF˙^–^ is further proved by CV and spectroelectrochemistry (Fig. S6 and S7†). From the rate constants of dimerization and disproportionation^37^ of PF˙^–^ we can estimate the rate constant for electron transfer (*k*_red_ ∼ 5 × 10^9^ s^–1^ M^–1^, Fig. S46†). The photoreduction with solvated electrons generated by photo-ionization of PF occurs at a rate close to the diffusion limit.^37^ The d^7^ complex [Cp*Rh^II^(bpy)H_2_O]^+^ created after the one electron reduction is not stable and disproportionates fast (*k*_disp_ = 3.7 × 10^8^ s^–1^ M^–1^)^33^ to a rhodium(i) complex. This d^8^ complex, [Cp*Rh^I^(bpy)], is then protonated†
†*k*_prot_ = 1.6 × 10^6^ s^–1^ M^–1^; U. Kölle; M. Grätzel, *Angew. Chem.*, 1987, **99**, 572. by a protic solvent to give Rh(iii)–H. In case of a possible direct hydride transfer between [Cp*Rh^III^(bpy)H_2_O]Cl_2_ and PFH_2_, Rh(iii)–H is formed directly.

Rh(iii)–H can either reduce the corresponding carbonyl (productive reaction) or can be protonated again to produce dihydrogen regenerating the catalyst.‡
‡Protonation: *k*_dec_ = 1.8 × 10^3^ s^–1^ M^–1^; *Angew. Chem.*, 1987, **99**, 572; reduction: *k*_red_ ∼ 2 × 10^3^ s^–1^ M^–1^. In case of the hydride reduction the carbonyl group is reduced to an alkoxy ligand, which is easily hydrolyzed^22^ giving the respective alcohol.

To investigate the fate of PF in the solution we examined the photoproducts formed from PF. The irradiation of degassed solutions of PF (*c* = 6.67 mmol) and TEOA (*c* = 133 mmol) provided a mixture of 2 photoproducts. The spectral characterization is provided in the ESI (Fig. S27 and S29†). A product absorbing at 340 nm was assigned to “leuco PF” whereas the second product absorbing at 424 nm was assigned to “diacridine” in accordance with published data.^38^ The first product is only observed when irradiating a degassed sample, whereas the second product is oxygen insensitive. Therefore we assume that leuco PF is formed from PF˙^–^ (triplet reductive pathway) and diacridine is formed from PF˙^+^ (singlet ionization pathway).

**Fig. 5:**
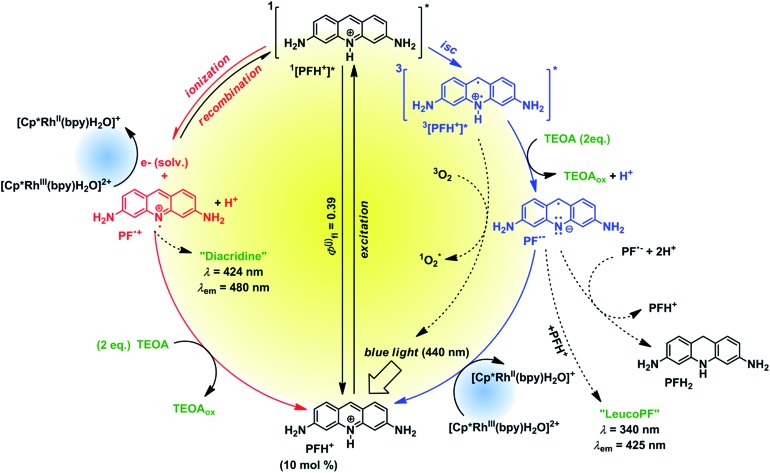
Proposed proflavine catalytic cycle.

Based on our mechanistic experiments and literature reports we propose the overall catalytic mechanism depicted in Fig. 5. After absorption of a blue photon PFH^+^ is excited to the first excited singlet state. Fluorescence (prompt and delayed) is a significant deactivation pathway with an overall quantum yield of 39%.^28^ Intersystem crossing (isc) gives the triplet state which accepts an electron from TEOA. The radical PFH˙ is deprotonated to the radical anion PF˙^–^, which is then oxidized by Rh_cat_ back to PFH^+^. In the absence of the metal complex the radical anion forms leuco PF and disproportionates to PFH_2_.^37^ The reduced Rh_cat_ reacts according to the catalytic cycle depicted in Fig. 4.

The control experiment without degassing (Table S1,† line 6) unexpectedly gave 30% of the product. As oxygen can efficiently quench both ^3^[PFH^+^]* and PF˙^–^ (for the rate constants see Fig. S45†), the product cannot be formed in this case through the triplet reductive pathway (Fig. 5, right side). We propose that another, oxygen-insensitive, pathway is present. PF is known for its photoionization from ^1^[PFH^+^]*§
§And partially (10%) from ^3^[PFH^+^]*; *Chem. Phys. Lett.*, 1980, **69**, 61–65. after excitation.^31^ Pileni and Grätzel^31^ reported that the photoionization is a single-photon process, whereas Hussein and Goez examined the process in more detail and revealed that the photoionization is caused by multiple photon process (*i.e.* absorption of the excited state).^39^ The photoionization produces solvated electrons^40^ which react¶
¶*k*_red_ = 2.5 × 10^10^ s^–1^ M^–1^; S. Solar; W. Solar; N. Getoff, *Z. Naturforsch., A: Phys., Phys. Chem., Kosmophys.*, 1982, **37**, 1077. either with PFH^+^ to form PF˙^–^ or with‖
‖*k*_red_ ∼ 10^10^ s^–1^ M^–1^; estimated value, based on: S. Solar; W. Solar; N. Getoff, *Z. Naturforsch., A: Phys., Phys. Chem., Kosmophys.*, 1982, **37**, 1077. Rh_cat_ to form Rh(ii) species.^37^ Unlike the triplet pathway, the PET from the singlet state is obviously an outer-sphere process. The oxidized PF radical cation [PF˙]^+^ is than reduced back by TEOA present in the system.**
**Redox potential of [PFH˙]^2+^ is *E*_0_ = +1.07 V *vs.* SCE, Fig. S6.† Δ*G*° = –*e* × (–0.76 V + 1.07 V) – 0.08 eV = –0.39 eV ∼ –37 kJ mol^–1^. These two parallel mechanisms (oxidative and reductive quenching) have been recently found in an iridium-based photocatalytic system.^41^

**Fig. 6:**
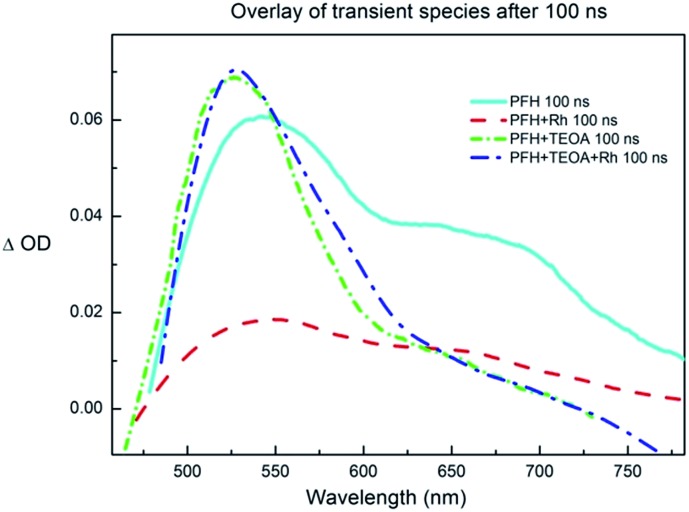
The overlay of the transient signal of proflavine (*c* = 2.24 × 10^–4^ M), TEOA (*c* = 2.58 × 10^–2^ M) and rhodium catalyst (*c* = 2.0 × 10^–4^ M) in DMF/water 1 : 1, bubbled with nitrogen, excitation wavelength *λ*_ex_ = 355 nm; time window 50 ns, 10× accumulated, 100 ns after the pulse, smoothed; the single peak at ∼530 nm corresponds to PF˙^–^ and the peak at ∼550 nm with a broad shoulder at ∼670 nm corresponds to ^3^[PFH^+^]*.

To further prove our mechanistic proposal, we performed a series of experiments using transient pump-probe spectroscopy (Fig. 6 and S32–S36†). The solution of PF (*c* = 2.2 × 10^–4^ M) in DMF/water mixture exhibited a strong fluorescence negative peak directly after the excitation flash (Fig. S32†). After ∼50 ns, when the fluorescence decays (the fluorescence lifetime was reported to be ∼5 ns)^31^ three peaks were observed at 550, 610 and 670 nm, respectively (Fig. 6). This was assigned to the ^3^[PFH^+^]*. The lifetime of the PF triplet was approx. 2 μs in aerated solution. The triplet spectrum and lifetime corresponds to the previously published data.^40^ The solution of PF and Rh_cat_ (*c*_Rh_ = 2.0 × 10^–4^ M) showed partially quenched fluorescence and the intensity of the PF triplet peak was significantly lowered (Fig. S34†). This finding corresponds to the Stern–Volmer experiment discussed previously and indicates that Rh_cat_ partially quenches the excited singlet state, which also leads to a diminished triplet yield.

The transient spectra of the solution of PF and TEOA (*c*_TEOA_ = 25.8 × 10^–3^ M) exhibited a new peak with an absorption maximum at ∼530 nm and with a lifetime of approx. 8 μs in aerated solution (Fig. S33†). The observed peak was oxygen-sensitive and corresponds to the PF˙^–^,^40^ confirming the PET from TEOA to ^3^[PFH^+^]*.

The transient spectrum of the solution of PF, TEOA and Rh_cat_ exhibited the absorption peak of PF˙^–^ (Fig. S35†). The intensity of the peak was lower than in the case of PFH^+^ and TEOA solution and its lifetime shortened to ∼3 μs caused by the electron transfer from the PF˙^–^ to Rh_cat_.

Rh_cat_ itself does not exhibit any transient species and no product from PET with TEOA is detected. Unlike its iridium analogue, Rh(iii)–H is not photoactive.^42^

The quantum yield of the product formation was determined to be *Φ* = (0.14 ± 0.05)% at 455 nm measured at low light intensity (*P*_absorbed_ = 9.3 mW, see ESI† for details). The low quantum yield is caused by loss of excitation by fluorescence (∼39%),^28^ low triplet yield (∼10%),^29^ disproportionation of the Rh^II^ species (two moles of PF˙^–^ for one mole of Rh^I^)^33^ and partial Rh(iii)–H decomposition (∼50% of Rh(iii)–H lost to H_2_).

## Conclusions

In summary, the selective photocatalytic reduction of aldehydes over ketones was achieved employing *in situ* generated Rh(iii)–H as the reduction reagent. Contrary to a formate-based aqueous reduction, the Rh(iii)–H is formed in the photocatalytic protocol slowly and allows therefore to kinetically distinguish between aldehydes and ketones. The photoreduction proceeds both *via* photoinduced electron transfer from the proflavine triplet and by oxidative quenching with Rh_cat_. The former pathway is oxygen sensitive and the latter is light intensity dependent. The light intensity influences directly the reaction mechanism and the reaction rate. A change of the light source (high-power LED *vs.* fluorescence light bulb) affects the product yield and the photocatalytic mechanism.
