# Supplementary material for: Visible light photocatalytic reduction of aldehydes by Rh(iii)–H: a detailed mechanistic study
Source: Chem Sci. 2015 Jan 6;6(3):2027–34. doi: 10.1039/c4sc03709j (PMC5654342; doi:10.1039/c4sc03709j)
Supplement: Supplementary file 1 [file SC-006-C4SC03709J-s001.pdf]

Electronic Supplementary Material (ESI) for Chemical Science.
This journal is © The Royal Society of Chemistry 2015

# Supplementary Materials for

## Visible light photocatalytic reduction of aldehydes by Rh(III)–H: A detailed mechanistic study

Tamal Ghosh,[†a] Tomáš Slanina[†a,b,c] and Burkhard König*[a]

[a] Institute of Organic Chemistry, University of Regensburg, D-93040 Regensburg, Germany.

[b] Department of Chemistry, Faculty of Science, Masaryk University, Kamenice 5, 62500 Brno, Czech Republic.

[c] Research Centre for Toxic Compounds in the Environment, Faculty of Science, Masaryk University, Kamenice 5, 62500 Brno, Czech Republic.

* To whom correspondence should be addressed: Burkhard.Koenig@ur.de

† These authors contributed equally to this work.

**Table of Contents**


General Methods and Material ... 3
General procedure for photocatalytic reductions ... 7
Product yields determination ... 7
Irradiation in a flow reactor ... 7
General procedure for the preparation of alcohols as standards ... 8
1-(4-(Hydroxymethyl)phenyl)ethanone ... 8
Methyl 4-(1-hydroxyethyl)benzoate ... 9
Preparation of [Cp*Rh(bpy)Cl]Cl ... 9
Control experiments ... 10
LED intensity check ... 11
Quantum yield determination ... 13
General procedure for chemical reductions ... 14
Chemical reductions – results ... 14
Imine reductions ... 15
Reduction potential measurements ... 17
Hydrogen production ... 21
Rhodium decomposition – reactivity model ... 23
pKa titration ... 26
Titration of proflavine with TEOA ... 29
Fluorescence studies ... 30
Preparation of RhIII hydride and its characterization ... 39
Irradiation of proflavine ... 41
Transient spectroscopy ... 45
References ... 53
NMR Spectra and Others ... 53

General Methods and Material

Proflavine (3,6-diaminoacridine hydrochloride, 95% dye content, catalogue number 131105-25G) was purchased from Sigma Aldrich. For all reactions and measurements proflavine was used in a form of hydrochloride. All other commercially available reagents and solvents were purchased and used without further purification.

Thin-layer chromatography was performed using silica gel plates 60 F254: Visualization was accomplished with short wavelength UV light (254 nm) and/or staining with appropriate stains (anisaldehyde, orthophosphomolybdic acid, $KMnO_4$).

Standard flash chromatography was performed on an Isolera™ Spektra Systems automated with high performance flash purification system using silica gel of particle size 40−63 μm. Macherey-Nagel silica gel 60 M (230-440 mesh) was used for column chromatography.

$^{1}H$ and $^{13}C$ NMR spectra were recorded on Bruker Avance spectrometers (300 MHz and 75 MHz or 400 MHz and 101 MHz) in $CDCl_3$ solution with internal solvent signal as reference (7.26 and 77.0, respectively). Proton NMR data are reported as follows: chemical shift (ppm), multiplicity (s = singlet, d = doublet, t = triplet, q = quartet, dd = doublet of doublets, ddd = doublet of doublets of doublets, td = triplet of doublets, m = multiplet, br. s. = broad singlet), coupling constants (Hz) and numbers of proton. Data for $^{13}C$ NMR are reported in terms of chemical shift and no special nomenclature is used for equivalent carbons.

Gas chromatography (GC) and gas chromatography coupled to low resolution mass spectrometry (GC-MS) analyses were performed using a capillary column (length: 30 m; diam. 0.25 mm; film: 0.25 μm) using He gas as carrier. GC was equipped with a FID detector. GC-MS was performed on 5975 MSD single quadruple detector. Reduction products were identified by comparing with authentic samples (GC/FID). Quantification of reduction products was performed by GC/FID analysis using internal standard. Head-space GC was performed on the Inficon Micro GC 3000 with a 3 Å mol sieve column, a thermal conductivity detector and Ar as carrier gas. The gas phase was taken directly from the sealed vial with the reaction mixture.

UV–Vis analyses were performed with Varian Cary 50 UV/Vis spectrophotometer and Agilent 8453 UV-Vis Spectrometer. Fluorescence measurements were performed with Horiba FluoroMax-4 fluorimeter. For UV and fluorescence measurements 10 mm Hellma fluorescence quartz cuvettes (117.100F-QS) with a screw cap with PTFE-coated silicon septum were used.

The UV-Vis measurements with online irradiation were performed on a self-made apparatus using a fluorescence cuvette in a fluorescence cuvette holder, LED (Cree-XP, royal blue, 455 nm) placed perpendicular to the optical pathway of the Agilent 8453 UV-Vis Spectrometer (Figures S1 and S2). The whole system was stirred with a small magnetic PTFE stirring bar by a magnetic stirrer placed above the cuvette.

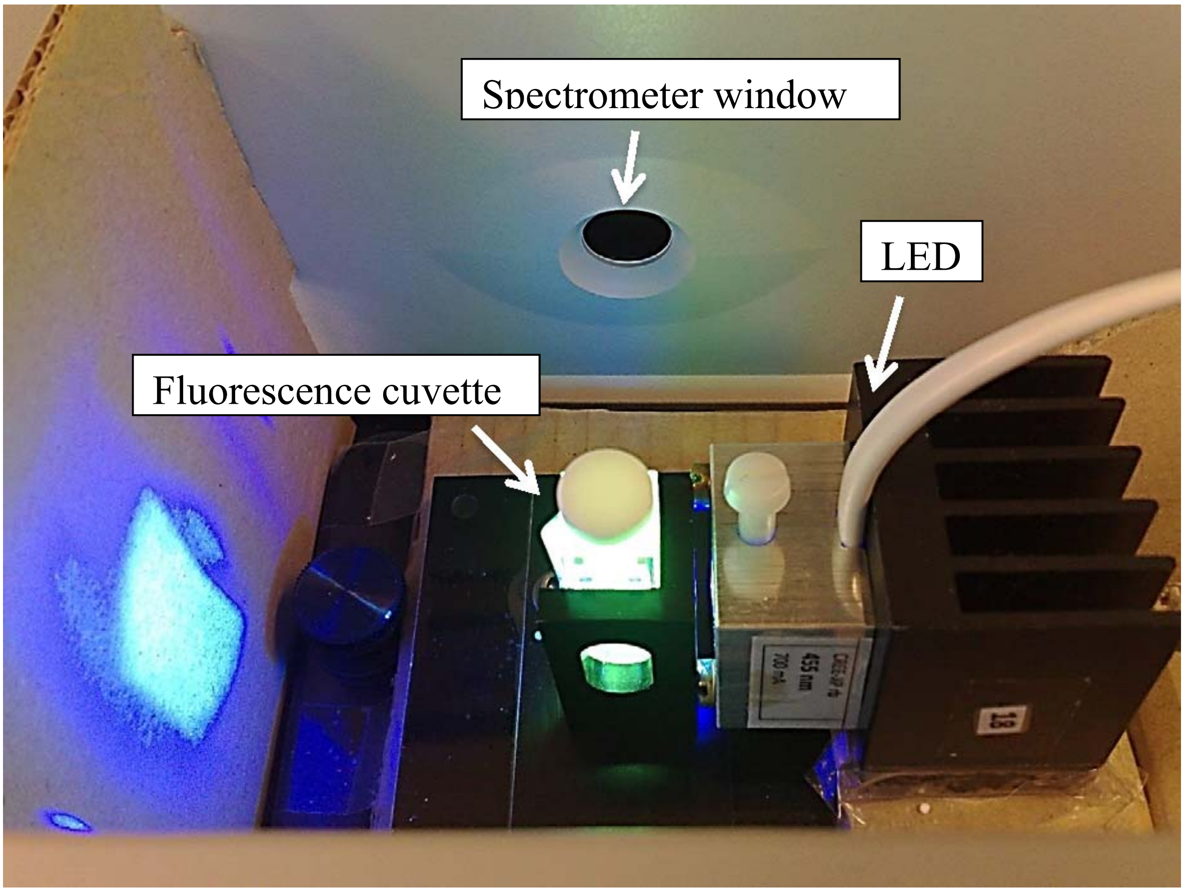


**Figure S1**: Setup for UV-Vis measurement with online irradiation

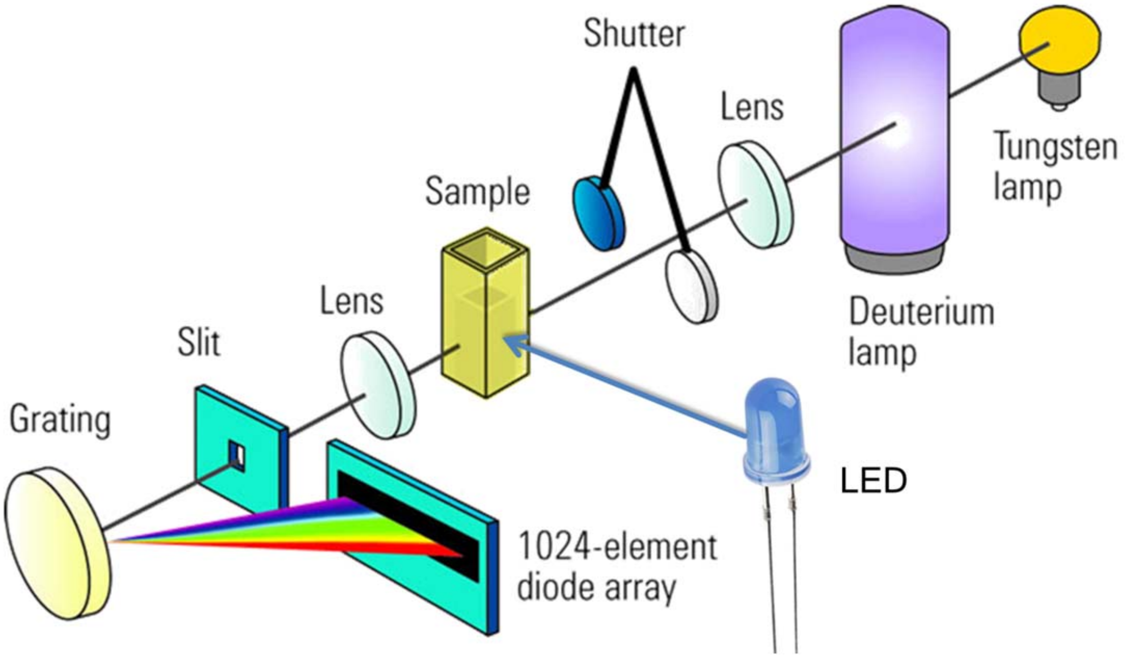


**Figure S2**: Scheme of the setup for UV-Vis measurement with online irradiation

Electrochemical studies were carried out under argon atmosphere. The cyclic voltammetry (CV) measurements were performed in acetonitrile (MeCN) containing 0.1 M tetra-n-butylammonium hexafluorophosphate as a conducting salt using ferrocene/ferrocenium ($Fc/Fc^{+}$) as an internal standard. A glassy carbon electrode (working electrode), platinum wire counter electrode, and Ag wire quasi-reference electrode were employed. The CV measurements of proflavine aqueous solutions were performed without conducting salt and internal standard. The Ag/AgCl electrode was used as a reference electrode instead of silver wire pseudoreference electrode. Spectroelectrochemical studies were carried out in an optically transparent thin layer electrochemical cell (OTTLE, 0.2 mm optical length). The Pt minigrid working electrode, Pt minigrid reference electrode and silver wire pseudoreference electrode were used.

Photocatalytic reactions were performed with 455 nm LEDs (OSRAM Oslon SSL 80 royal-blue LEDs, $\lambda_{em}$ = 455 nm (± 15 nm), 3.5 V, 700 mA).

For the reaction with a flow reactor 8 × LED OSRAM Oslon LDH9GP deep blue, 455 nm (Datasheet: http://tinyurl.com/n8vucd5) were used as a light source. The reaction mixture was injected by syringe pump (Landgraf Laborsysteme, Spritzenpumpe LA-100, RS232, Datasheet: http://tinyurl.com/p99nv3m) into a glass flow reactor (Figure S3, LTF factory, 11 × 5.7 cm, 1.7 mL internal volume, 0.3 mL volume of the reactor tubing) previously flushed with nitrogen. The

flow reactor was cooled down to 20°C with a custom-made aluminum cooling block placed opposite to LEDs with a mirror to minimize losses of the light.

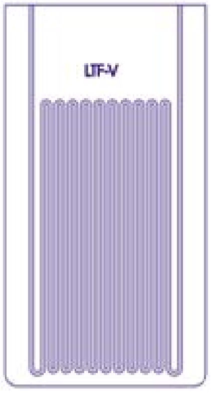


**Figure S3**: Flow reactor LFT-V

The quantum yield of the photoreduction was determined using the apparatus previously developed in cooperation with our group.[1] The 3 W 455 nm LED (LXHL-LR3C) was used for irradiation. The calibrated solar cell was used to measure precisely the output power of the light source. The measured value was in the linear range of the calibration curve. The background light was minimized by working in dark. The photoproduct yield was determined by GC/FID by internal standard method.

The pH measurements were accomplished with WTW pMX 3000 pH meter with glass pH electrode (Metrohm) and temperature sensor (WTW TFK 150).

The laser flash photolysis (LFP) setup was operated in a right-angle arrangement of the pump and probe beams. Laser pulses of ≤ 700 ps duration at 355 nm (170 mJ) were obtained from an Nd:YAG laser and were dispersed over the 4 cm optical path of the quartz cell by a concave cylindrical lens.[2] The absorbance of the sample solutions was adjusted to 0.3 − 0.5 per cm at the wavelength of excitation. A 75 W xenon lamp was used as the source of white probe light. Spectrographic detection (ICCD camera connected to a spectrometer equipped with 300 l/mm gratings blazed at 300 or 500 nm) of the transient absorptions was available. A fresh solution was used for each laser flash to avoid excitation of the photoproducts. Measurements were done at ambient temperature (20 ± 2 °C). All samples were degassed by bubbling with nitrogen directly in the cuvette.

All UV-Vis and fluorescence spectra were measured in aqueous solutions unless mentioned otherwise.

General procedure for photocatalytic reductions

The carbonyl compound (0.1 mmol, 1 eq.), triethanolamine (29.8 mg, 0.2 mmol, 2 eq.), proflavine (2.5 mg, 0.01 mmol, 10 mol%), rhodium catalyst (4.7 mg, 0.01 mmol, 10 mol%) were mixed with a small PTFE stirring bar in a 5 mL crimp cap vial. The vial was sealed with a PTFE septum and DMF/water mixture (1:1, v/v, 1.5 mL) was added. The resultant solution was degassed by 3 freeze-pump-thaw cycles and filled with nitrogen atmosphere.
The reaction vessel was placed in a cooling block cooled to 20 °C, was irradiated through the plane bottom side by 3W blue LED ($\lambda_{em}$ = 455 nm) and the reaction conversion was monitored by GC analysis. The samples for GC were taken by a degassed gas-tight Hamilton syringe. After complete conversion an internal standard (*e. g.* ethyl benzoate, 0.5 mL, $c$ = 20 mg/mL) was added to the reaction mixture and the product yield was determined by GC/FID using a calibrated method.

Product yields determination

All photoreduction products were compared with authentic standards either purchased (Sigma Aldrich, Acros) or synthetized according to the published procedures and compared with known spectra. In most cases the mass balance between the substrate and the product determined from the calibration curve corresponded to the conversion.

Irradiation in a flow reactor

Reaction in the flow reactor decreased the irradiation time. The 1 mm flow reactor tubing was irradiated from both sides and therefore all volume of the reaction mixture was irradiated. To keep the system degassed, the flow reactor was first flushed with nitrogen for 10 min and then it was filled with degassed solvent mixture. The degassed reaction mixture was filled into a 5 mL syringe (internal diameter = 13 mm) which was connected to the syringe pump. The reaction mixture was expelled through the tubing to the flow reactor so that the front of the reaction solution was right at the beginning of the irradiated part of the tubing. The syringe pump was set to the flow rate 0.5 mL/h, corresponding to 203 min irradiation time of the reaction mixture

(irradiated volume = 1.7 mL). The flow rate was optimized by decreasing the flow rate and measuring the product yield.

General procedure for the preparation of alcohols as standards

The reduction reactions were performed according to known procedures.[3] Sodium borohydride (46 mg, 1.23 mmol, 1.1 eq.) was added to a stirred solution of carbonyl compound (1.12 mmol, 1 eq.) in freshly distilled EtOH (10 mL). The course of the reaction was monitored by TLC (EtOAc:hexane = 1:5) and the reaction was complete after 5 minutes. After stirring for 10 minutes the reaction mixture was concentrated *in vacuo* and the residue dissolved in DCM (30 mL). The organic layer was subsequently washed with ammonium chloride (30 mL, sat. aq.), distilled water (30 mL) and brine (30 mL), the organic extract dried over $MgSO_4$ and concentrated in vacuo to afford the respective alcohol (yield: 57–91%).

1-(4-(Hydroxymethyl)phenyl)ethanone

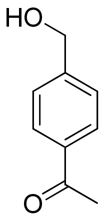


The product was prepared according to the modified general procedure. In order to prevent the double reduction the sodium borohydride was dissolved in EtOH (5 mL) and the substrate solution cooled to 0 °C was titrated by this solution with TLC check after each addition. The reduction was complete after addition of 0.288 eq. (~1.15 reduction equivalents) of $NaBH_4$. The workup followed the general procedure. The crude product was purified by column chromatography (EtOAc:hexane = 1:1) yielding a white crystalline solid. The spectroscopic data match previously reported values.[4]

Yield: 153 mg (57%)

$^1$H-NMR: $\delta_H$ (300 MHz, $CDCl_3$): 7.96 (d, $J$ = 8.4 Hz, 2H), 7.46 (d, $J$ = 8.6 Hz, 2H), 4.79 (s, 2H), 2.61 (s, 3H), 1.61 (br. s., 1H).

$^{13}$C-NMR: $\delta_C$ (75 MHz, $CDCl_3$): 198.07, 146.27, 136.31, 128.65, 126.64, 64.60, 26.68.

Methyl 4-(1-hydroxyethyl)benzoate

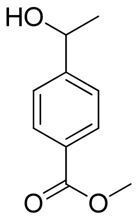

The product was prepared according to the modified general procedure. The reaction in ethanol yielded the transesterification product [ethyl 4-(1-hydroxyethyl)benzoate]. Freshly distilled methanol was used for the reaction yielding colorless oil. The spectroscopic data match previously reported values.[5]

Yield: 164 mg (91%)

$^{1}$H-NMR: $\delta_H$ (300 MHz, $CDCl_3$): 8.02 (d, $J$ = 8.4 Hz, 2H), 7.45 (d, $J$ = 8.6 Hz, 2H), 4.97 (q, $J$ = 6.5 Hz, 1H), 3.93 (s, 3H), 2.31 (s, 1H), 1.52 (d, $J$ = 6.5 Hz, 3H).

$^{13}$C-NMR: $\delta_C$ (75 MHz, $CDCl_3$): 167.05, 151.03, 129.84, 129.10, 125.31, 69.93, 52.14, 25.30.

Preparation of [Cp*Rh(bpy)Cl]Cl

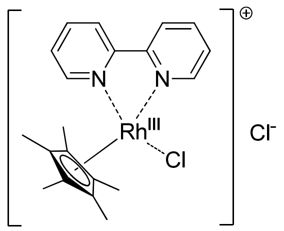

The compound was prepared according to a known procedure.[6] Rhodium dimer $[RhCl_2Cp^*]_2$ (100 mg, 0.162 mmol, 1 eq) was suspended in methanol (4 mL) and 2,2'-bipyridyl (61 mg, 0.388 mmol, 2.4 eq.) was added. The solid rhodium complex dissolved in 5 minutes. The orange-yellow solution was stirred for 20 min at room temperature. The solvent was concentrated to about 2 mL, the product precipitated by adding diethyl ether (10 mL) while stirring, filtered by a Büchner

funnel, washed with diethyl ether (2 × 5 mL) and dried under reduced pressure yielding 150 mg (>99%) of an orange powder.

Yield: 0.150 g (>99%)

Melting point: 180°C, decomposition

$^1$H-NMR: $\delta_H$ 9.04 (d, $J$ = 8.0 Hz, 2H), 8.84 (d, $J$ = 5.6 Hz, 2H), 8.26 (td, $J$ = 8.0, 1.5 Hz, 2H), 7.81 (td, $J$ = 8.0, 1.5 Hz, 1H), 1.74 (s, 15H).

Control experiments

All control experiments were repeated three times. For each control experiments one component of the reaction system depicted in Scheme S1 was omitted or substituted (Table S1).

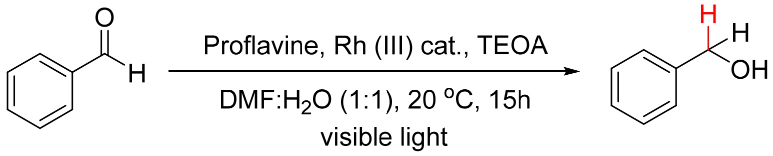


**Scheme S1**: Typical reaction procedure

**Table S1**: Control experiments

| Entry | Proflavine (mol%) | Rh (III) cat. (mol%) | TEOA (eq.) | Light (455 nm) | Atmosphere | Yield (%)[a] |
|---|---|---|---|---|---|---|
| Control experiments | | | | | | |
| **1** | **10** | **10** | **2** | **Yes** | **$N_2$** | **97** |
| 2 | 10 | 10 | - | Yes | $N_2$ | 0 |
| 3 | 10 | - | 2 | Yes | $N_2$ | ~0 |
| 4 | - | 10 | 2 | Yes | $N_2$ | ~0 |
| 5 | 10 | 10 | 2 | - | $N_2$ | 0 |
| **6** | **10** | **10** | **2** | **Yes** | **Air** | **30** |
| 7 | 10 | - | 2 | Yes | $H_2$ | 0 |
| 8 | 10 | 10 | 2 | - | $H_2$ | 0 |

[a] GC/FID determined yield with appropriate internal standard

<u>LED intensity check</u>

The reaction yield of identical reactions shows some variation (±10 %). Having established a reproducible degassing procedure the relative intensities of different LEDs were investigated. Irradiation was accomplished in a cooling block with six positions for gas tight vials (Figure S4).

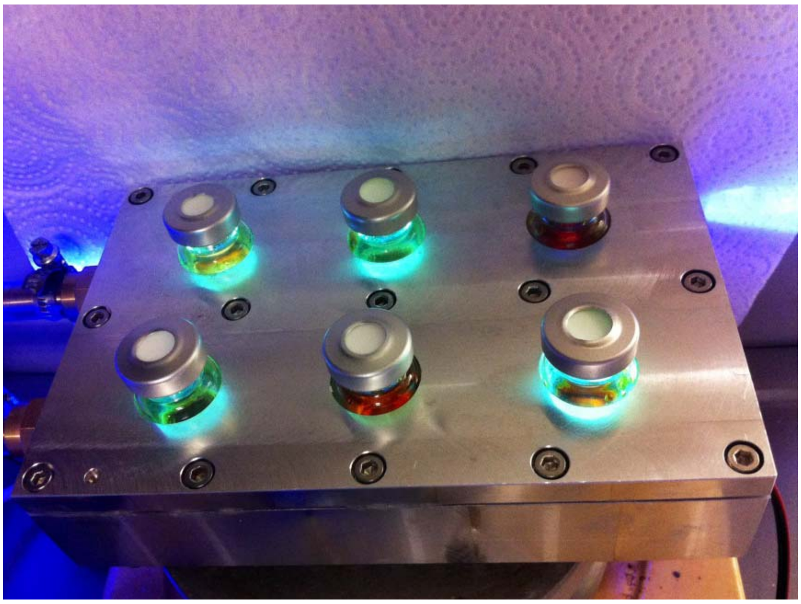

**Figure S4**: Irradiation setup

Despite the fact that all LEDs in one cooling block were of the same age and had the same voltage (parallel connection), the intensity of emitted light differed for each position. The results of the test reaction are summarized in Figure S5.

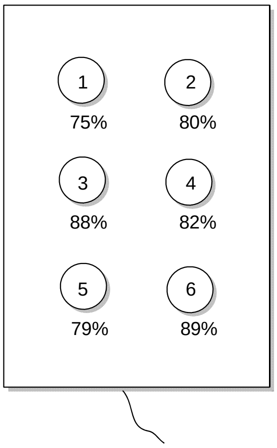


**Figure S5**: LED intensity test of different positions of the cooling block; the respective yields of benzaldehyde reduction are shown below each position

Quantum yield determination

The quantum yield of the photocatalytic reduction of benzaldehyde (typical reaction) was determined by a method developed in cooperation with our group.[1] The reaction mixture (3 mL, DMF/water 1:1) of benzaldehyde ($c$ = 5 mM), TEOA ($c$ = 10 mM), rhodium catalyst ($c$ = 0.5 mM), proflavine hydrochloride ($c$ = 0.5 mM) and internal standard for GC ($c$ = 5 mg/mL) was filled into a fluorescence cuvette with a stirring bar and septum and degassed by bubbling by nitrogen (20 min).

The measurement was accomplished in a dark room to minimize the ambient light. The radiant power of light transmitted by the cuvette with a blank solution was measured ($U_{\mathrm{ref}}$ = 96 mV) and the transmitted power ($P_{\mathrm{ref}} = U_{\mathrm{ref}}/10$ = 9.6 mW) was noted. The cuvette with blank was changed by cuvette with the reaction mixture and the transmitted radiant power ($P_{\mathrm{sample}}$ = 0.3 mW) was noted. The transmitted radiant power was monitored during the irradiation and remained constant.

The sample was irradiated for 14 hours to reach 17% conversion (0.277 mg, $2.56 \times 10^{-6}$ mol; the LED power was significantly lower than in the photocatalytic setup).

The quantum yield was calculated from Equation S1:

$$\Phi = \frac{N_{product}}{N_{ph}} = \frac{N_{\mathrm{A}} * n_{product}}{\frac{E_{light}}{E_{ph}}} = \frac{N_{\mathrm{A}} * n_{product}}{\frac{P_{absorbed} * t}{\frac{h * c}{\lambda}}} = \frac{h * c * N_{\mathrm{A}} * n_{product}}{\lambda * \left(P_{ref} - P_{sample}\right) * t} \quad \text{(S1)}$$

where $\Phi$ is quantum yield, $N_{product}$ is the number of molecules created, $N_{ph}$ is the number of photons absorbed, $N_{\mathrm{A}}$ is Avogadro's constant in moles$^{-1}$, $n_{product}$ is the molar amount of molecules created in moles, $E_{light}$ is the energy of light absorbed in Joules, $E_{ph}$ is the energy of a single photon in Joules, $P_{absorbed}$ is the radiant power absorbed in Watts, $t$ is the irradiation time in sec, $h$ is the Planck's constant in J×s, $c$ is the speed of light in m s$^{-1}$, $\lambda$ is the wavelength of irradiation source (455 nm) in meters, $P_{ref}$ is the radiant power transmitted by a blank cuvette in Watts and $P_{sample}$ is the radiant power transmitted by the cuvette with reaction mixture in Watts.

This results in:

$$\Phi = \frac{h * c * N_A * n_{product}}{\lambda * (P_{ref} - P_{sample}) * t}$$

$$= \frac{6.626 \times 10^{-34}\text{Js} \times 2.998 \times 10^{8}\text{ms}^{-1} \times 6.022 \times 10^{23}\text{mol}^{-1} \times 2.56 \ \times 10^{-6}\text{mol}}{455 \times 10^{-9}\text{m} \times (9.6 - 0.3) \times 10^{-3}\text{Js}^{-1} \times 50400\text{s}}$$

$$= \frac{3.062 \times 10^{-7}\text{Jm}}{2.133 \times 10^{-4}\text{Jm}} = 1.44 \times 10^{-3} \cong \mathbf{0.14}\ \%$$

From 3 independent measurements the quantum yield was determined to be $\Phi = (0.14 \pm 0.05)\%$.

General procedure for chemical reductions

The reductions were accomplished according to the known procedure.[7] A mixture of carbonyl compound (0.5 mmol, 1 eq.) and rhodium catalyst (1.15 mg, 2.5 µmol, 0.5 mol%) in formate buffer (2 mL, 2M, pH = 3.50) was degassed in a 5 mL sealed glass vial by bubbling with nitrogen and stirred. The reaction mixture changed color from yellow to deep blue as the rhodium(III) hydride was formed in the system. Formic acid reacts with the catalyst generating gases ($H_2$, $CO_2$) and therefore a balloon with nitrogen was attached to the vial.

Aliquots (50 µL of reaction mixture) were taken and extracted by ethyl acetate (1.0 mL) in an Eppendorf tube. The organic extract was measured by GC monitoring the reaction kinetics.

Chemical reductions – results

The results of chemical reductions are summarized in the Table S2. The limiting factor of many organic substrates is the poor solubility/miscibility with the aqueous formate buffer. The reactions with sodium or ammonium formate as a formate ion source did not yield the reduction product.

**Table S2**: Chemical reductions

| Entry | Substrate | Reaction time (min) | Conversion |
|---|---|---|---|
| 1 | cyclohexane | 120 | 100 |
| 2 | benzaldehyde | 15 | 100 |
| 3 | acetophenone | 15 | 40 |
| 4 | benzaldehyde/acetophenone | 15 | 94/32[a] |

[a] conversion of benzaldehyde/acetophenone

Imine reductions

Analogous to the carbonyl reductions imines were reduced in various solvents (see Table S3) and when noted, thiourea (1 eq.) was used as additive. Imine reductions are shown in Scheme S2. The reduction product 1-amino diphenylmethane was determined by GC-FID by spiking with a purchased authentic sample. The procedure (*e. g.* loading of reagents, degassing procedure) was analogous to carbonyl reductions. Water had to be excluded from the system because otherwise the hydrolysis was a dominant reaction pathway.

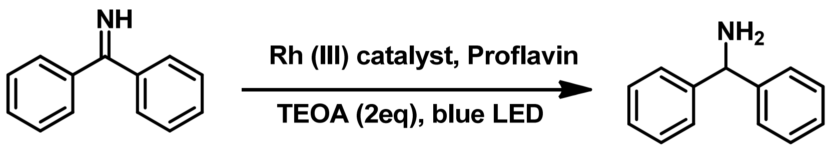


**Scheme S2**: General scheme of imine reductions

The results of reductions are summarized in Table S3.

**Table S3**: Imine reductions

| Entry | Reaction conditions | Time (h) | Yield (%)[a] |
|---|---|---|---|
| 1 | $Ph_2C$=NH, $CH_3CN$, 20 °C | 15 | 11 |
| 2 | $Ph_2C$=NH, $CH_3CN$, 20 °C, thiourea | 15 | 87 |
| 3 | $Ph_2C$=NH, DMF, 20 °C, thiourea | 5.5 | 63 |
| 4 | $Ph_2C$=NH, DMF, 20 °C, thiourea | 17 | 84 |
| **5** | **$Ph_2C$=NH, DMSO, 20 °C, thiourea** | **5.5** | **81** |
| 6 | $Ph_2C$=NH, DMSO, 20 °C, thiourea | 17 | 86 |
| 7 | $Ph_2C$=NH**.**HCl, $CH_3CN$, 20 °C, thiourea | 17 | 47 |

[a] uncalibrated yield based on GC/FID

Reduction potential measurements

The reduction potentials were measured by cyclic voltammetry (CV). The half-wave potential of the first reduction peak was taken as the first reduction potential of the compound. The scan-rate was kept at 50mV/s and the average value from tree scans was taken.

The cyclic voltammogram is shown in the Figure S6. The potentials were determined to be $E_{red}$ = –1.16 V vs Ag/AgCl = –0.74 V vs SCE; $E_{ox}$ = +1.20 V vs Ag/AgCl = +1.07 V vs SCE.[a]

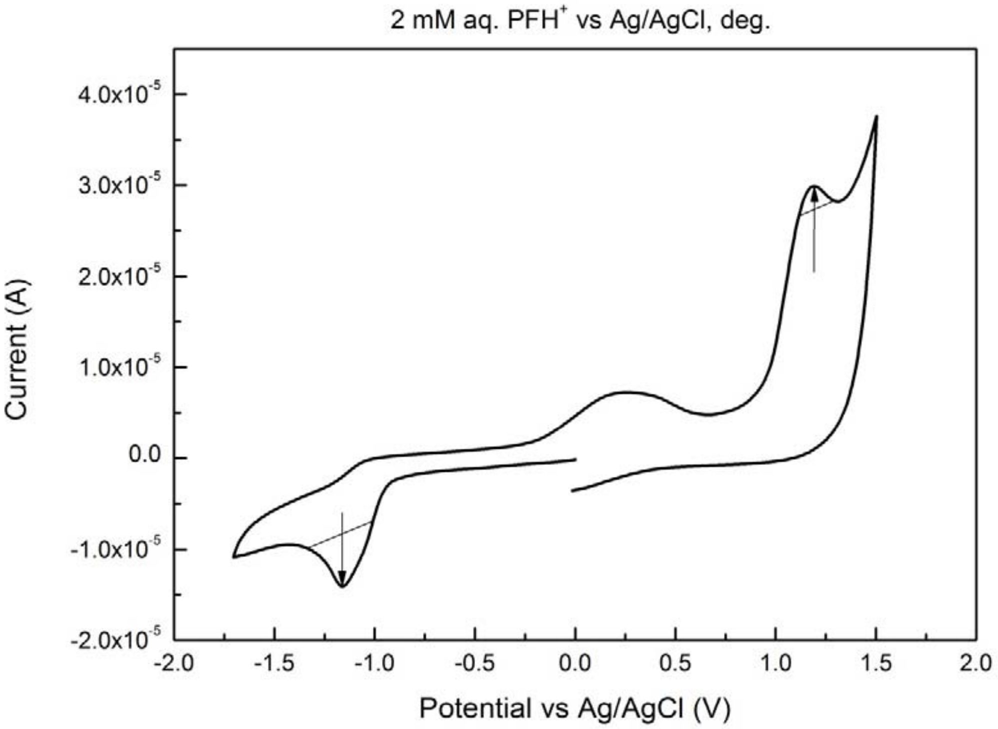


**Figure S6**: Cyclic voltammogram of proflavine hydrochloride in aqueous solution ($c$ = 2 mM). The reduction peak and oxidation peak are shown by arrows ($E_{red}$; $E_{ox}$).

The spectroelectrochemical data of reduction of aqueous solution of proflavine hydrochloride (2 mM, degassed) are shown in Figure S7. The UV-Vis spectra were measured every 100 mV. Both the cyclic voltammetry (irreversible reduction peak) and spectroelectrochemical data (appearance of the new peak at 393 nm) suggest the deprotonation after the reduction.

[a] according to Pavlishchuk, V. V.; Addison, A. W. *Inorg Chim Acta* **2000**, *298*, 97.

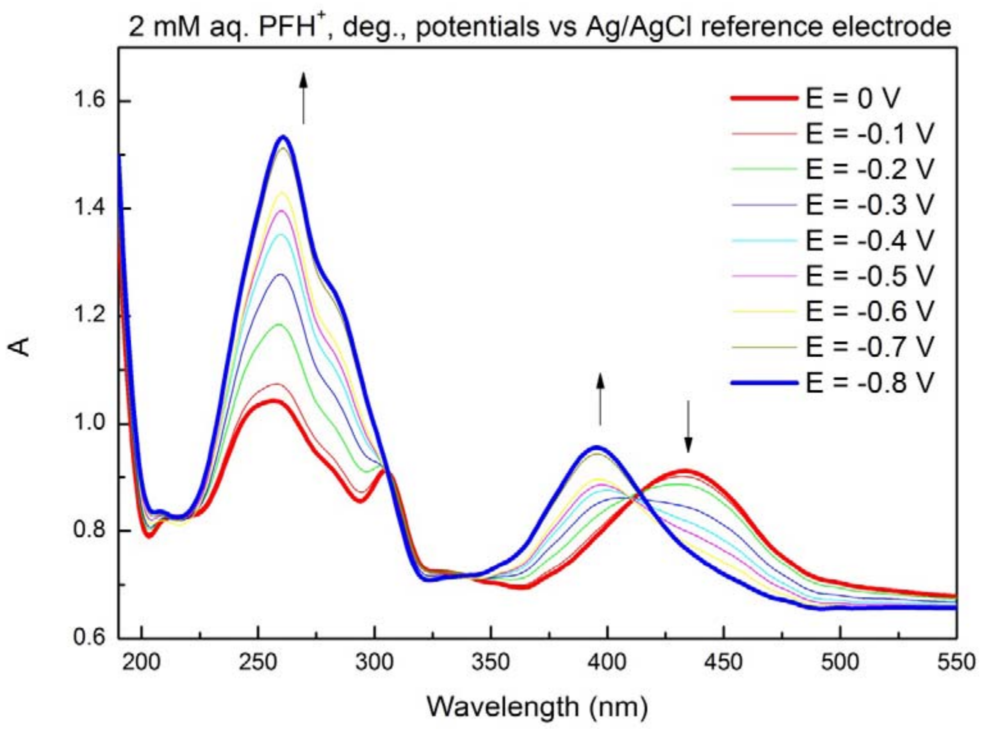


**Figure S7**: Spectroelectrochemical data of reduction of proflavine hydrochloride

The CV of a variety of carbonyl compounds was measured in order to make a library of reference data. All benzaldehydes exhibited a reversible reduction peak ($E_1$) whereas acetophenones showed two reduction peaks, first irreversible ($E_1$) and second reversible ($E_2$). When the cyano group was present in the molecule, an additional peak corresponding to the reduction of CN functionality was present in the voltammogram.

**Table S4**: Reduction potentials (all values are shown in V, the potentials were measured in acetonitrile with $BuN_4PF_6$ (0.1 mM solution) and ferrocene as an internal standard. The potentials of all reduction peaks are shown. The measured potentials were determined eith $Fe/Fe^+$ oxidation peak as internal standard and recalculated to SCE[b].

| No. | Compound | $E_1$ meas. | $E_1$ vs SCE | $E_2$ meas. | $E_2$ vs SCE | $E_3$ meas. | $E_3$ vs SCE |
|---|---|---|---|---|---|---|---|
| 1 | Benzaldehyde | –1.91 | –1.94[c] | | | | |
| 2 | 4-MeO-benzaldehyde | –2.30 | –2.37 | | | | |
| 3 | 4-MeO-acetophenone | –2.44 | –2.48 | | | | |
| 4 | Benzyl acetone | <–3.0 | <–3.1 | | | | |
| 5 | Acetophenone | –2.36 | –2.38[d] | | | | |
| 6 | 4-F-benzaldehyde | –1.96 | –2.03 | | | | |
| 7 | 4-Acetoxy-benzaldehyde | –1.43 | –1.47 | –2.01 | –2.06 | | |
| 8 | 4-CN-benzaldehyde | –1.45 | –1.59 | –2.75 | –2.90 | | |
| 9 | 4-CN-acetophenone | –1.64 | –1.84 | –2.20 | –2.41 | –2.71 | –2.92 |
| 10 | 3,4-Dimethoxy-benzaldehyde | –2.11 | –2.24 | | | | |
| 11 | 3,4,5-Trimethoxy-benzaldehyde | –1.91 | –2.02 | | | | |
| 12 | Methyl 4-acetylbenzoate | –1.84 | –1.84 | –2.22 | –2.2 | –2.70 | –2.70 |
| 13 | 4-$CF_3$ acetophenone | –1.98 | –2.11 | –2.45 | –2.58 | | |

[b] according to Pavlishchuk, V. V.; Addison, A. W. *Inorg Chim Acta* **2000**, *298*, 97.
[c] corresponds to the published value of -1.94 V vs SCE; *Handbook of Photochemistry* 2nd edition, NY, 1993
[d] corresponds to the published value of -2.38 V vs SCE; *Catal. Commun.* **2010**, 11, 1049-1053.

| | | | | | | | |
|---|---|---|---|---|---|---|---|
| 14 | Cyclohexyl carbaldehyde | <–3.0 | <–3.1 | | | | |
| 15 | 2,2,2-Trifluoro acetophenone | –1.66 | –1.65 | –2.28 | –2.27 | | |
| 16 | 2,4-Dimethoxy-benzaldehyde | –2.19 | –2.27 | | | | |
| 17 | Methyl 4-formylbenzoate | –1.76 | –1.71 | | | | |
| 18 | Fluorenone | –1.34 | –1.43 | –1.87 | –1.95 | | |
| 19 | Phenyl acetaldehyde | –3.00 | –2.89 | | | | |
| 20 | 3-Phenyl propionaldehyde | <–3.0 | <–3.1 | | | | |
| 21 | Phenylacetone | <–3.0 | <–3.1 | | | | |
| 22 | Cyclohexanone | <–3.0 | <–3.1 | | | | |

The measured reduction potentials were correlated with Hammett's sigma values[e] of the substituents on the aromatic ring. The correlation with the yields at respective reaction times is shown in Figure S8.

[e] C. Hansch and A. Leo, "Substituent Constants for Correlation Analysis in Chemistry and Biology," Wiley-Interscience, NY, 1979.

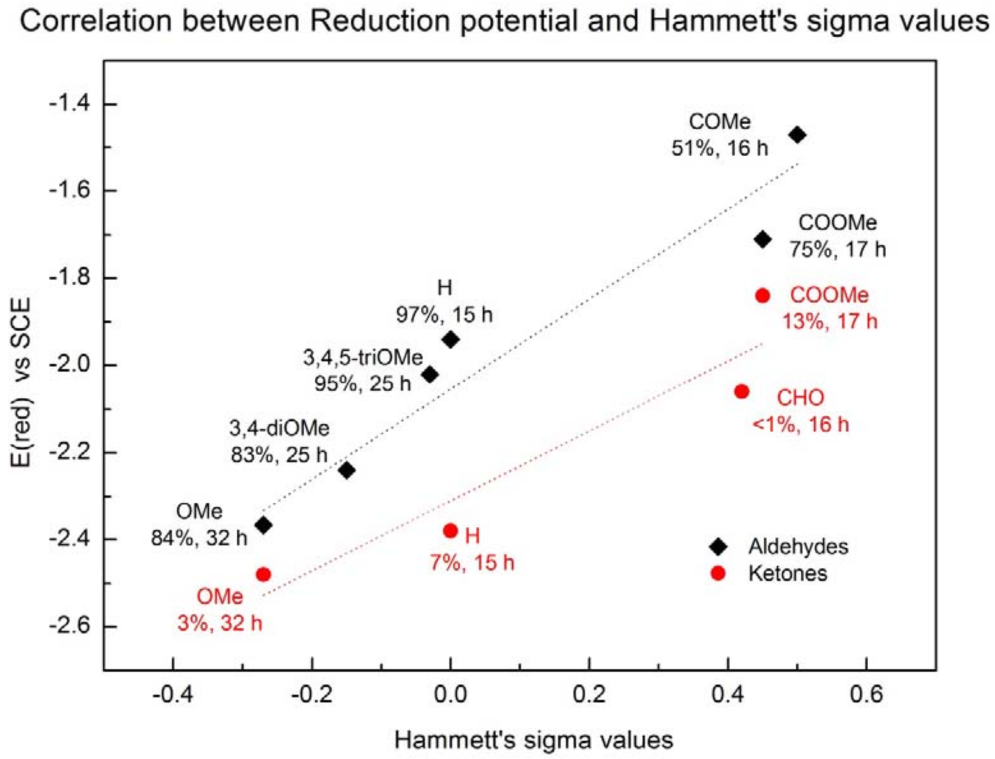


**Figure S8**: Correlation between reduction potentials and Hammett's sigma value[f] of aromatic aldehydes and acetophenones. The product yield at respective reaction time is shown.

Hydrogen production

The amount of hydrogen generated as a byproduct of the photoreduction was measured by head-space analysis. The detector response was calibrated to be linear dependent on the hydrogen concentration. The typical chromatogram is shown in Figure S9. The results of the hydrogen evolution in photoreduction of benzaldehyde and acetophenone are shown in Figure S10.

[f] C. Hansch and A. Leo, "Substituent Constants for Correlation Analysis in Chemistry and Biology," Wiley-Interscience, NY, 1979.

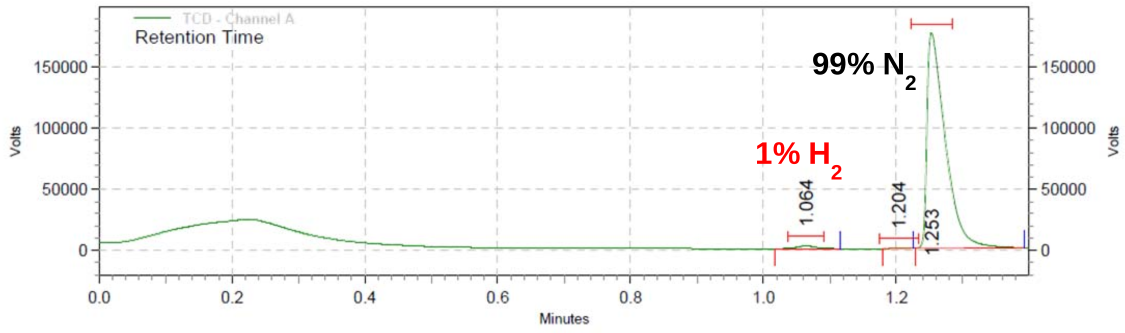


**Figure S9**: Typical head-space chromatogram; the hydrogen peak (ret. time $t_R$ = 1.064 min) and nitrogen peak (ret. time $t_R$ = 1.253 min) is shown. The peak at 1.204 min corresponds to the residual oxygen introduced at the injection.

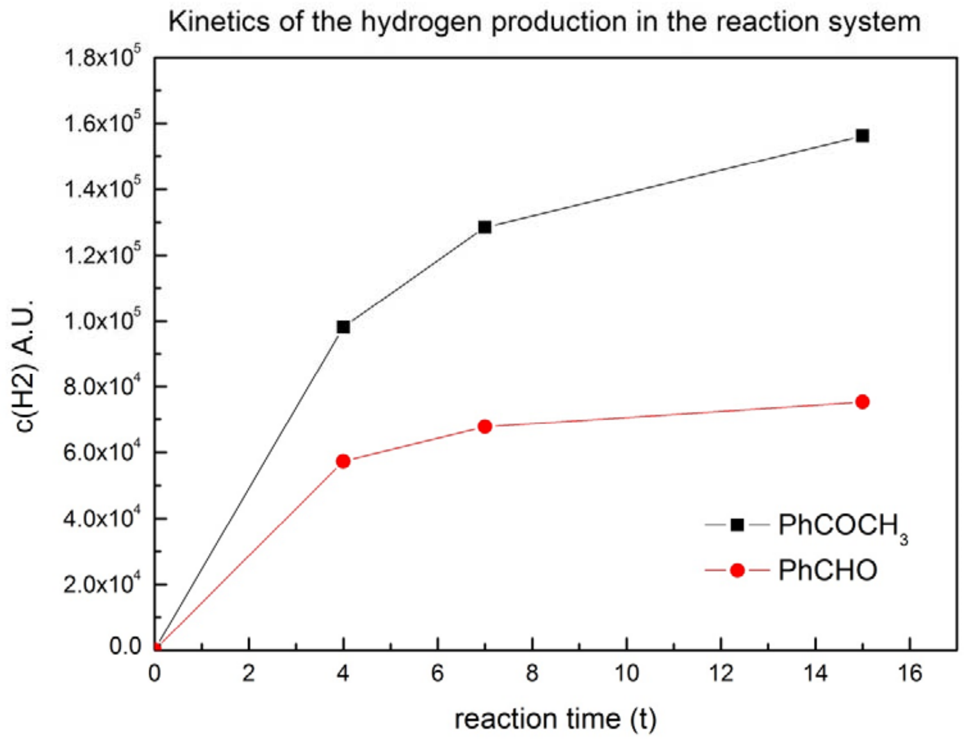


**Figure S10**: The kinetics of hydrogen production for the reaction with acetophenone (squares, black line) and benzaldehyde (closed circles, red line).

The kinetics of benzaldehyde photoreduction was followed by both GC-FID and Head-space GC. In both cases the result corresponded to the first order decay kinetics shown in Figure S11. The rate constant of benzaldehyde reduction was determined to be ~0.1537 $hr^{-1} = 4.27 \times 10^{-5}\ s^{-1}$.

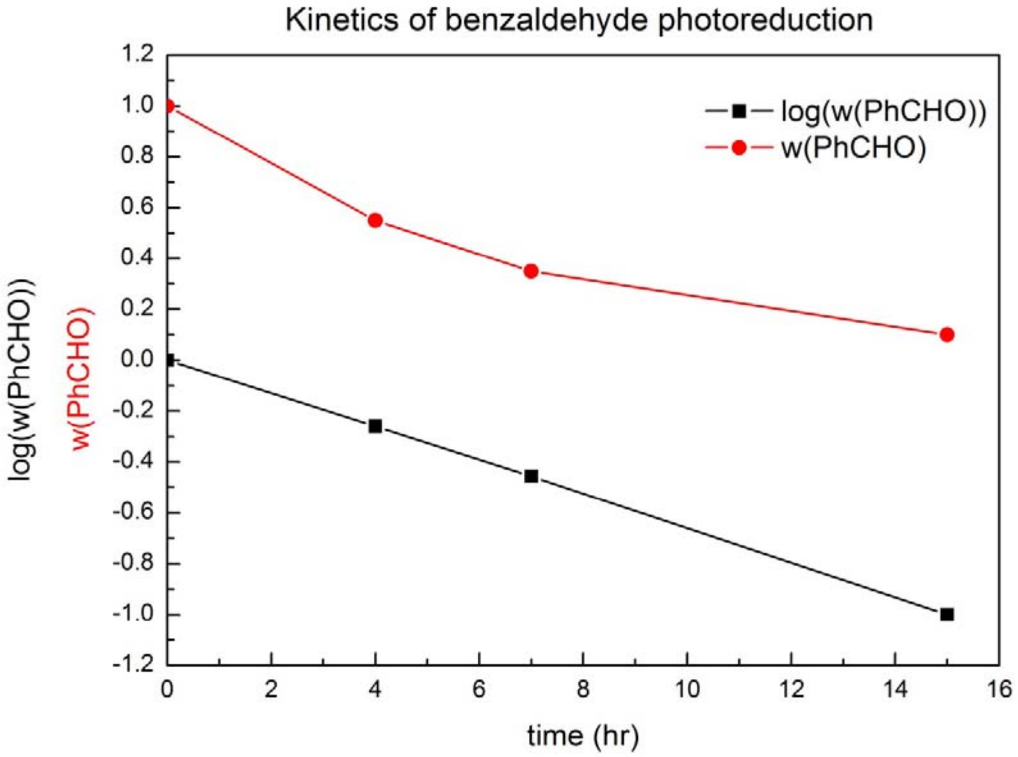


**Figure S11**: Kinetic evolution of benzaldehyde in photocatalytic reduction

<u>Rhodium decomposition – reactivity model</u>

The reactivity model of reaction of rhodium hydride with aldehyde and ketone present simultaneously in the reaction mixture was constructed. The general reactivity is shown in Scheme S3.

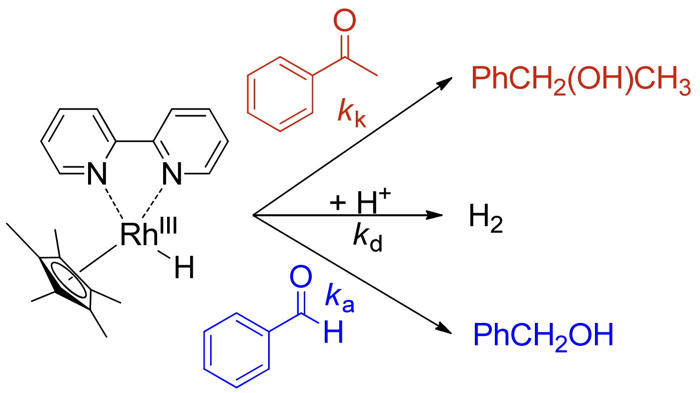


**Scheme S3**: General reactivity scheme of rhodium hydride with aldehyde and ketone in the reaction mixture

In case that all reactions in Scheme S3 are of first order (simplified model valid at low conversion), the resultant rate equations are summarized below.

$$\frac{d[\mathrm{RhH}]}{dt} = -(k_a + k_d + k_k)[\mathrm{RhH}]$$

$$[\mathrm{RhH}] = [\mathrm{RhH}]_0 \times e^{-(k_a+k_d+k_k)t}$$

$$\Phi_{ald} = \frac{k_a}{k_a + k_d + k_k}$$

$$\Phi_k = \frac{k_k}{k_a + k_d + k_k}$$

$$\text{selectivity: } \boldsymbol{s} = \frac{\Phi_{ald}}{\Phi_k} = \frac{\frac{k_a}{k_a + k_d + k_k}}{\frac{k_k}{k_a + k_d + k_k}} = \frac{\boldsymbol{k_a}}{\boldsymbol{k_k}}$$

In this case the aldehyde/ketone selectivity is time and conversion independent.

The more realistic model assumes all reductions to be of second order and the decomposition to be of pseudo first order. The rate constants are summarized below.

$$\frac{d[\mathrm{RhH}]}{dt} = -(k_a[\mathrm{RCHO}] + k_d[\mathrm{H}^+] + k_k[\mathrm{RCOR}])[\mathrm{RhH}]$$

$$\Phi_{ald} = \frac{k_a[\mathrm{RCHO}]}{k_a[\mathrm{RCHO}] + k_d[\mathrm{H}^+] + k_k[\mathrm{RCOR}]}$$

$$\Phi_k = \frac{k_k[\mathrm{RCOR}]}{k_a[\mathrm{RCHO}] + k_d[\mathrm{H}^+] + k_k[\mathrm{RCOR}]}$$

$$\text{selectivity}: \boldsymbol{s} = \frac{\Phi_{ald}}{\Phi_k} = \frac{\dfrac{k_a[\mathrm{RCHO}]}{k_a[\mathrm{RCHO}] + k_d[\mathrm{H}^+] + k_k[\mathrm{RCOR}]}}{\dfrac{k_k[\mathrm{RCOR}]}{k_a[\mathrm{RCHO}] + k_d[\mathrm{H}^+] + k_k[\mathrm{RCOR}]}} = \frac{\boldsymbol{k_a[\mathrm{RCHO}]}}{\boldsymbol{k_k[\mathrm{RCOR}]}}$$

$$\text{selectivity } t = 0: \boldsymbol{s_0} = \frac{k_a[\mathrm{RCHO}]_0}{k_k[\mathrm{RCOR}]_0} = \frac{\boldsymbol{k_a}}{\boldsymbol{k_k}}$$

The aldehyde/ketone selectivity is time and conversion dependent, it decreases with increasing conversion and is maximal at the beginning of the reaction.

The product development and the aldehyde/ketone selectivity dependence are shown in the Figure S12. Second order kinetic equations and the following constants were used: $k_a = 0.15\ \mathrm{hr}^{-1}$; $k_k = 0.0025\ \mathrm{hr}^{-1}$. The results after 16 hours (93 % conversion of aldehyde and 4 % of ketone) correspond well with the experimental data. The value $k_a = 0.15\ \mathrm{hr}^{-1}$ supports the result of head-space analysis (Figure S11).

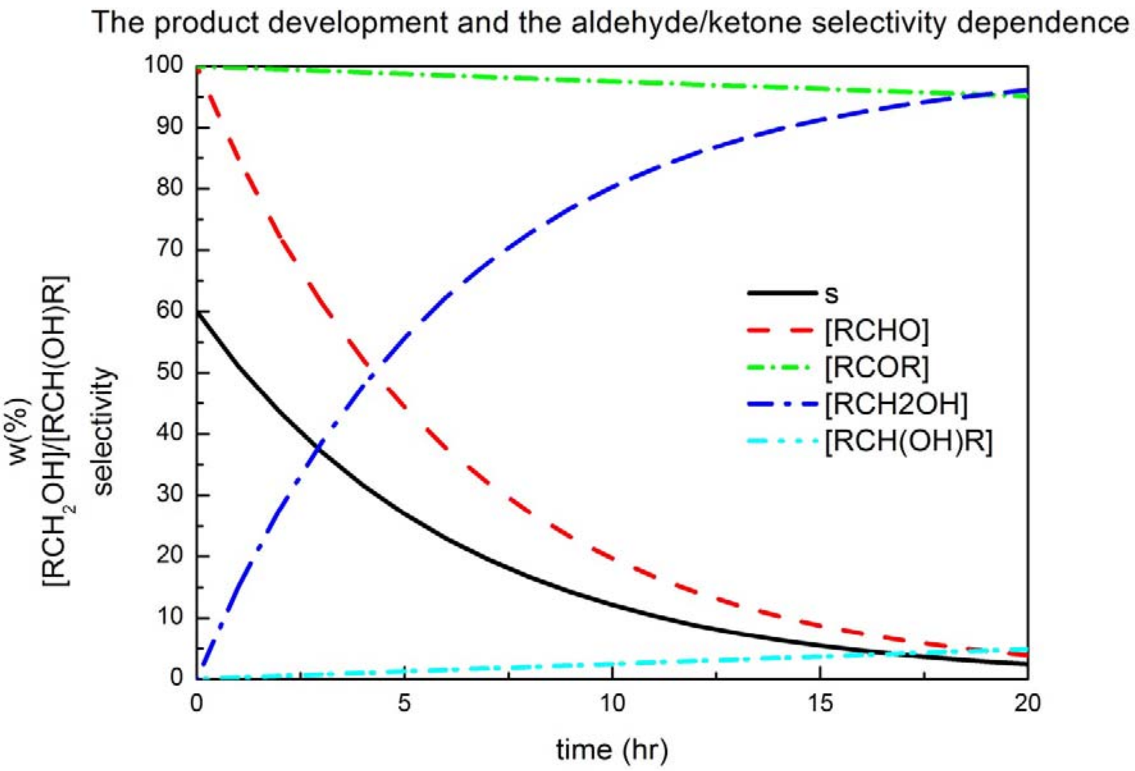


**Figure S12**: Model of the time dependence of the substrate and product molar ratio (in %; red, blue, green and cyan line) and selectivity (s, solid black line)

Figure S12 shows clearly that the selectivity drops dramatically with conversion and that the absolute selectivity (= 100% vs 0%) at full conversion for substrates reacting by side reactions with a common reagent is not possible.

pKa titration

The p*K*a of proflavine hydrochloride was determined by spectrophotometrical titration. The protonated form has a maximum at 443 nm whereas the neutral form has its maximum at 393 nm. The p*K*a of proflavine hydrochloride was determined to be 9.6 which corresponds to the literature data.[8] The titration profile is depicted in Figure S13. The fitted titration curves are depicted in Figure S14.

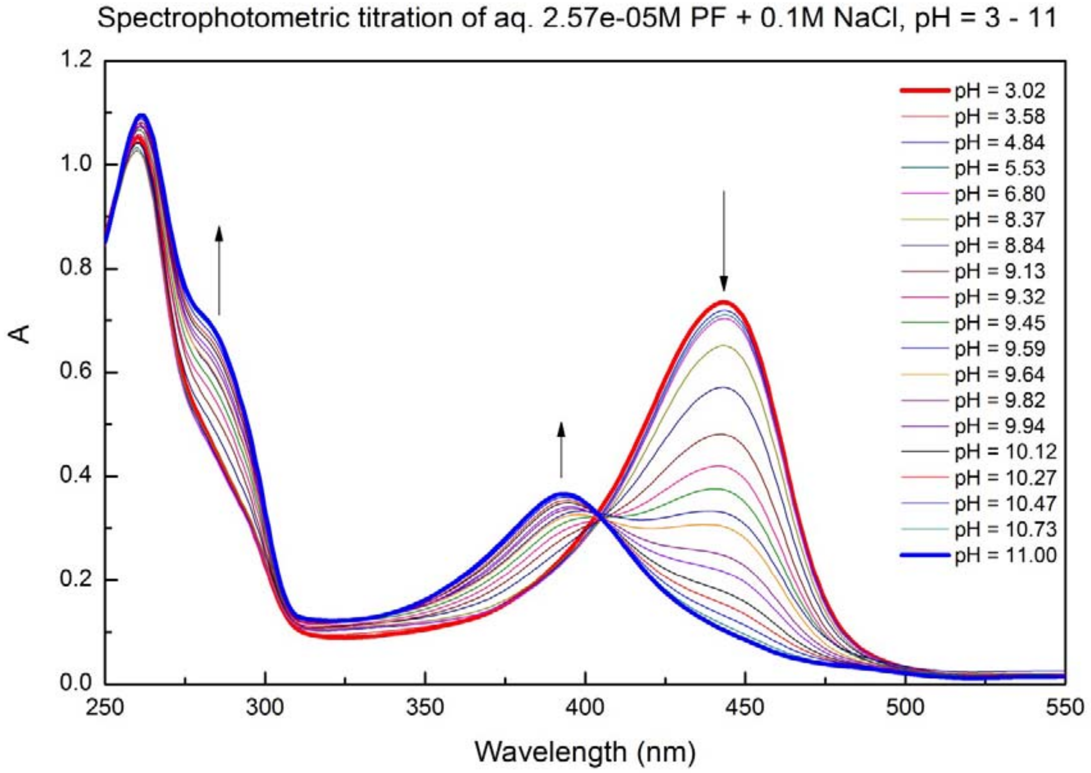


**Figure S13**: Spectrophotometrical titration of aq. proflavine hydrochloride ($c$ = 2.57 × $10^{-5}$ M) from pH = 3.0 to pH = 11.0, 0.1 M NaCl was used to keep the ion strength constant

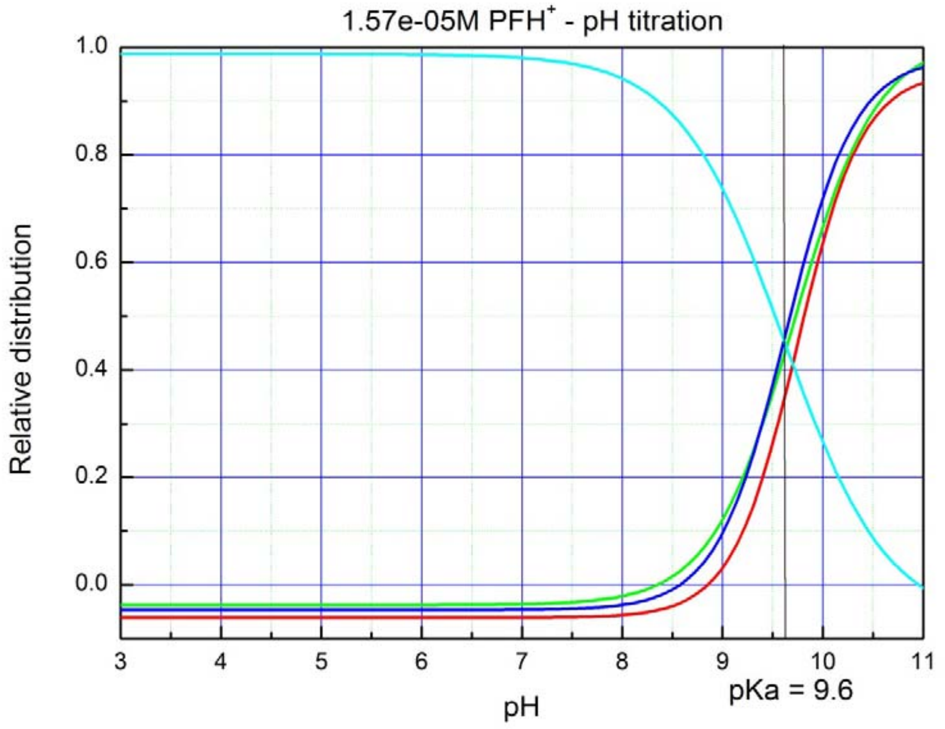


**Figure S14**: Titration curve of proflavine hydrochloride fitted from spectrophotometric titration, p*K*a is depicted by black vertical line

Titration of proflavine with TEOA

Proflavine hydrochloride was also titrated by TEOA and UV-Vis spectra were recorded. The same acidobasic behavior was observed as in case of p*K*a titration.

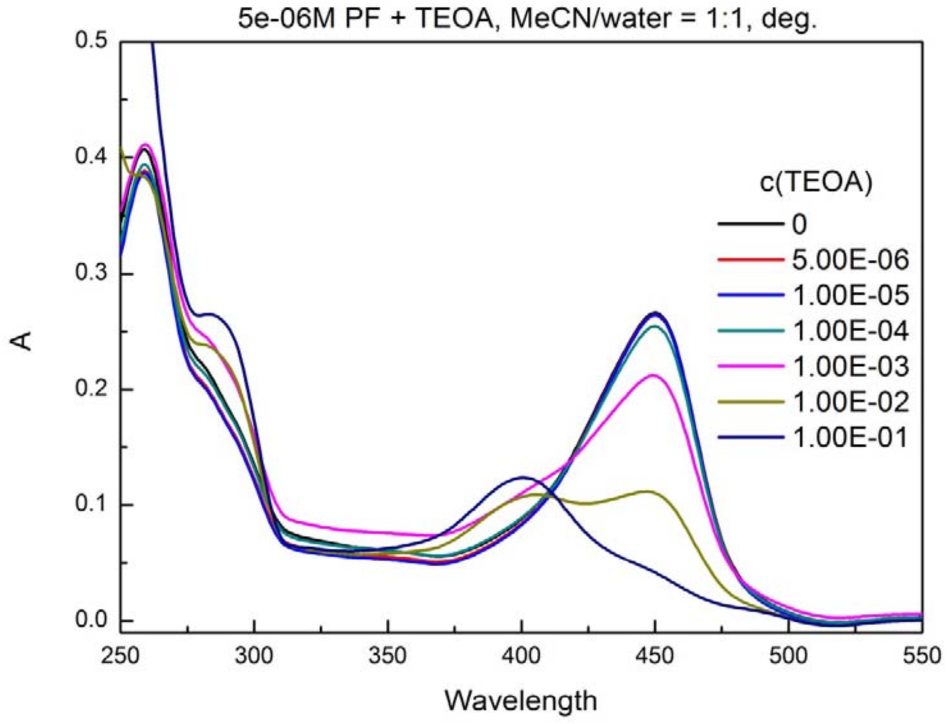


**Figure S15**: Titration of 5 × $10^{-6}$ M proflavine hydrochloride with TEOA in degassed acetonitrile/water 1:1 mixture

Fluorescence studies

The fluorescence pH titration of proflavine hydrochloride is shown in Figure S16.

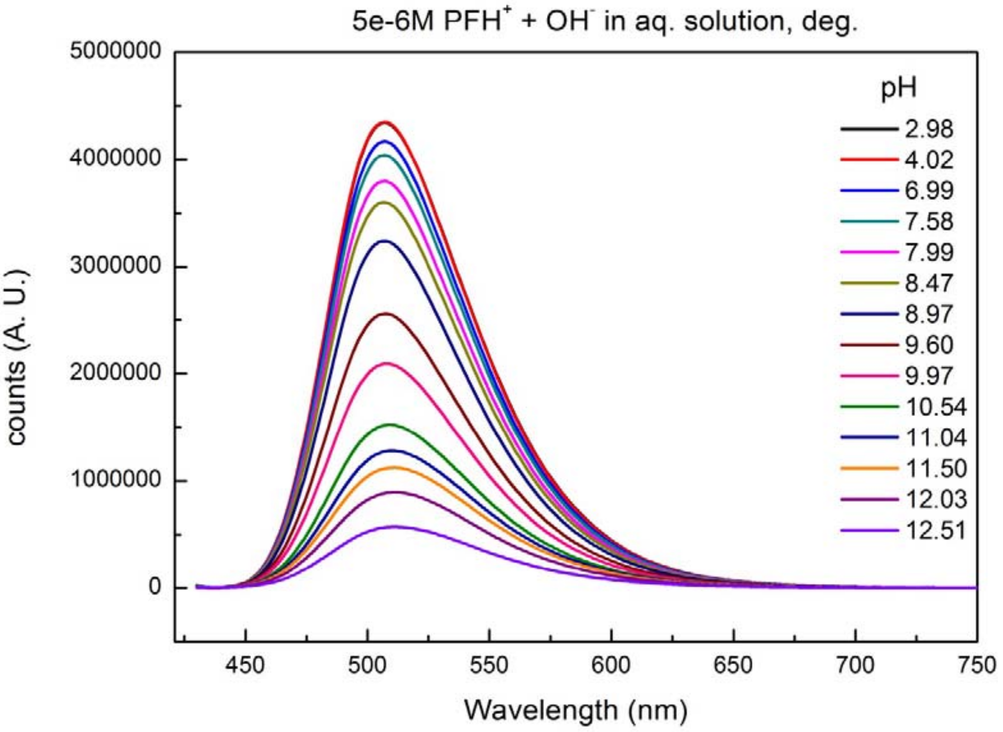


**Figure S16**: Fluorescence quenching of aq. proflavine hydrochloride ($c$ = 5 × $10^{-6}$ M) with hydroxide ion (pH = 3 – 12.5)

The titration curve from the fluorescence maximum ($\lambda$ = 508 nm) is shown in the Figure S17. The p*K*a of proflavine hydrochloride (p*K*a = 9.5) is shown with a vertical line as well as the p*K*a of excited singlet state (p*K*a = 12.5) were determined to be in inflection points of the curve. Both values correspond with the published data.[8,9]

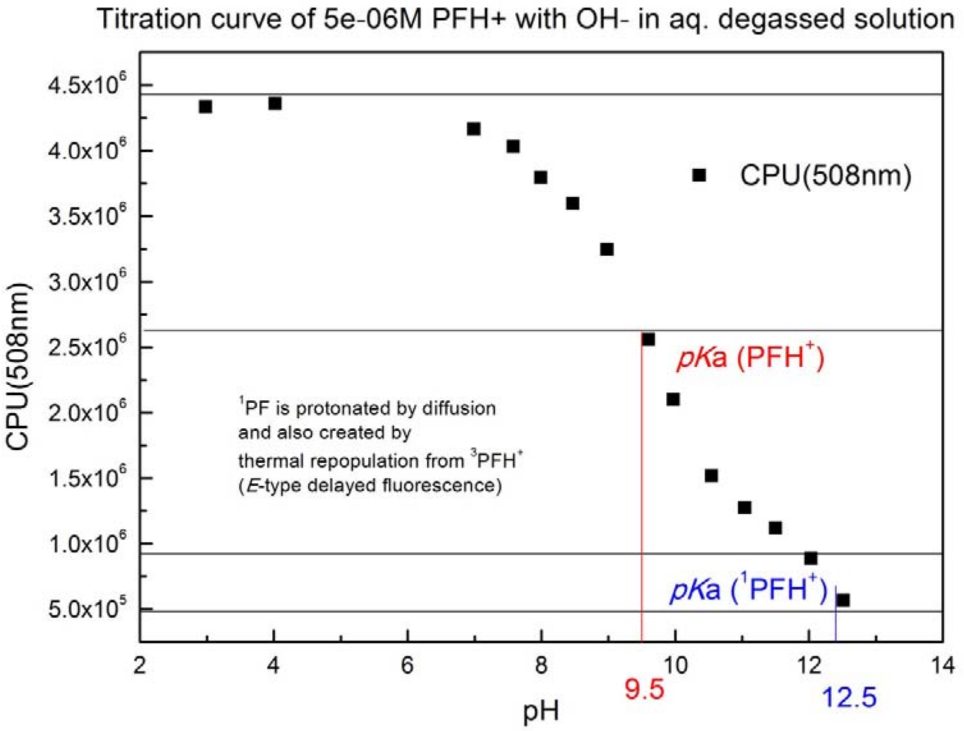


**Figure S17**: Fluorescence titration of proflavine hydrochloride with p*K*a values depicted

The fluorescence titration of proflavine hydrochloride with TEOA is shown in Figure S18.

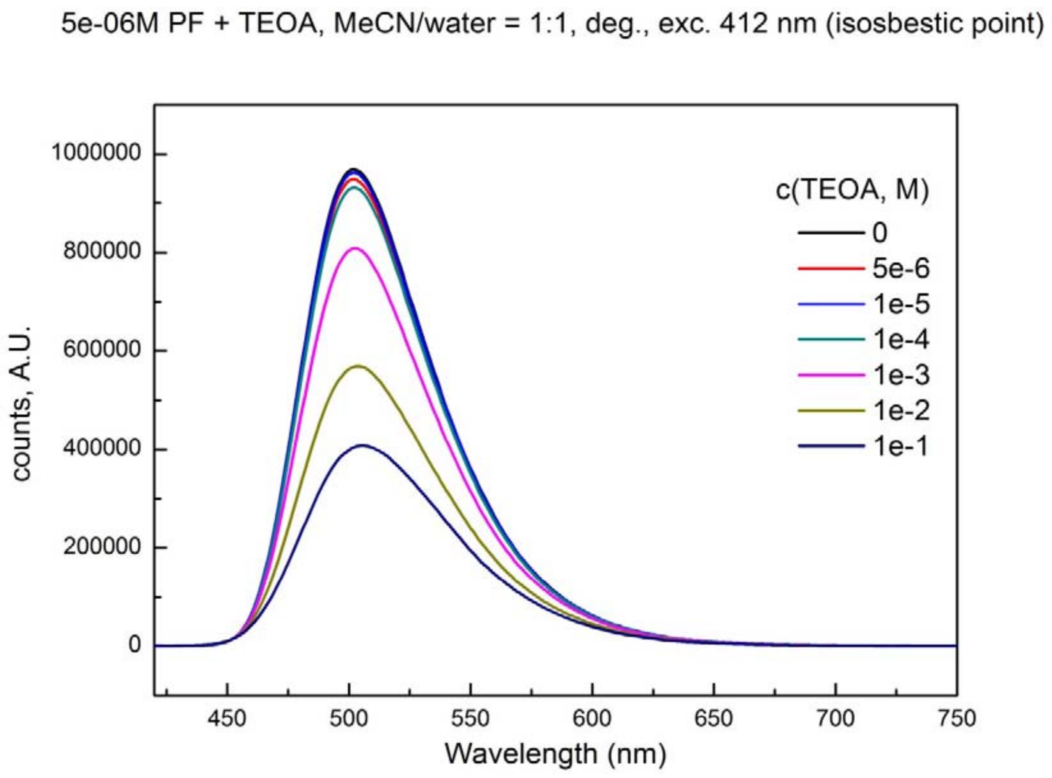


**Figure S18**: Fluorescence titration of proflavine hydrochloride with TEOA

The titration curve from the fluorescence maximum (λ = 508 nm) of TEOA and $OH^-$ is shown in the Figure S19. The equation S2 was determined experimentally. The last point of TEOA titration curve deviates from the pH titration because TEOA serves as a buffer with maximal buffer capacity at pH = 9.6 (p*K*a of $TEOAH^+$).

$$\mathrm{pOH} = -\log[\mathrm{TEOA}] + 2.5 \quad \mathrm{S2}$$

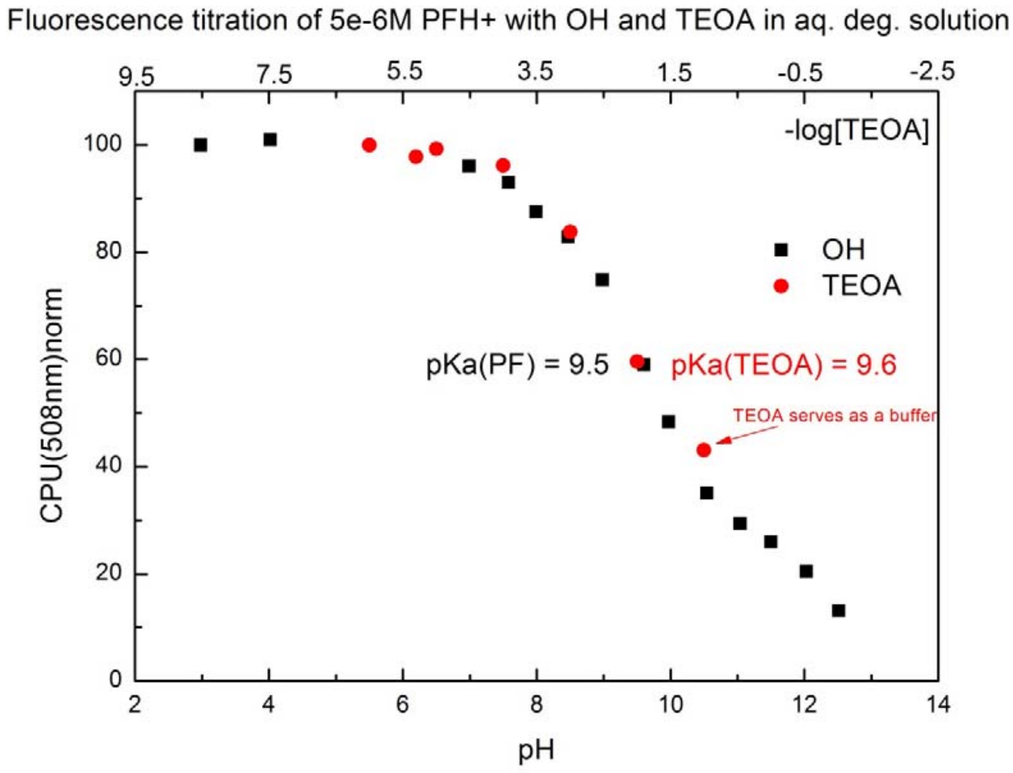


**Figure S19**: Comparison of titration curves of proflavine hydrochloride with $OH^-$ and TEOA

From fluorescence titration and TEOA UV-vis titration of proflavine hydrochloride the relative distributions of PF and $PFH^+$ were calculated according to equations S3 – S5.

$$\chi_{PFH^+} = 1 - \frac{I_{PFH} \quad - I_i}{I_{PFH} \quad - I_{PF}} \quad \text{S3}$$

$$A^{450} = \varepsilon^{PF(450)} \cdot \chi_{PF} + \varepsilon^{PFH(450)} \cdot \chi_{PFH} \quad \text{S4}$$

$$A^{401} = \varepsilon^{PF(401)} \cdot \chi_{PF} + \varepsilon^{PFH(401)} \cdot \chi_{PFH} \quad \text{S5}$$

where $\chi_{PFH}$ is the molar distribution of protonated proflavine, $\chi_{PF}$ is the molar distribution of neutral proflavine, $I_{PFH}$ is the fluorescence intensity (λ = 508 nm) of fully protonated proflavine, $I_{PF}$ is the fluorescence intensity (λ = 508 nm) of fully deprotonated proflavine, $I_i$ is the fluorescence intensity (λ = 508 nm) of the actual sample, $\varepsilon^{PF(450)}$ is the molar distribution coefficient of the neutral form of proflavine, $\varepsilon^{PF(450)}$ is the molar distribution coefficient of the protonated form of proflavine and $A^{450}$ resp. $A^{401}$ are absorbances at relative wavelengths.

The distribution calculated from the UV-Vis measurements correspond to the ground state acidobasic reaction and the distribution calculated from the fluorescence measurement correspond to the "quenching". The comparison of these two is shown in Figure S20. Since the inflexion point of both curves lies at the same concentration of TEOA, no fluorescence quenching was observed. The partial quench would be observed if the inflexion point calculated from fluorescence would be located at lower concentration TEOA than the one calculated from UV-Vis measurement.

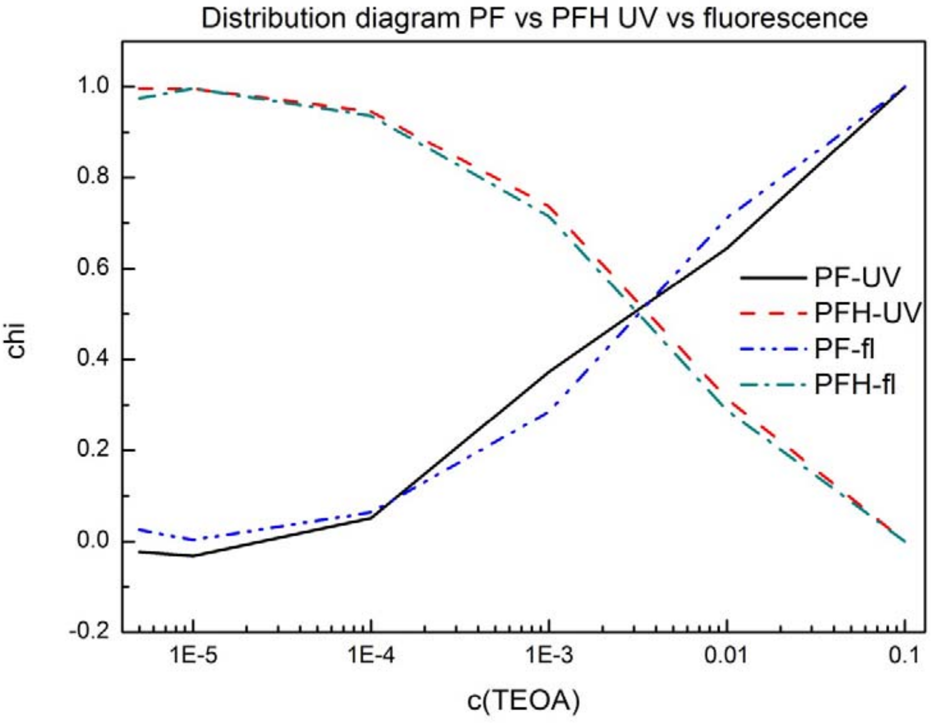


**Figure S20**: Distribution diagram of relative acidobasic forms of proflavine calculated from UV-Vis and fluorescence measurement

The Stern-Volmer analysis of fluorescence titration of proflavine hydrochloride either with $OH^-$ or TEOA is shown in Figure S21. Since the points do not lie on a straight line, the quenching is not observed in either case and the decrease of fluorescence is caused by acidobasic equilibrium between strongly fluorescent hydrochloride and weakly fluorescent neutral proflavine.

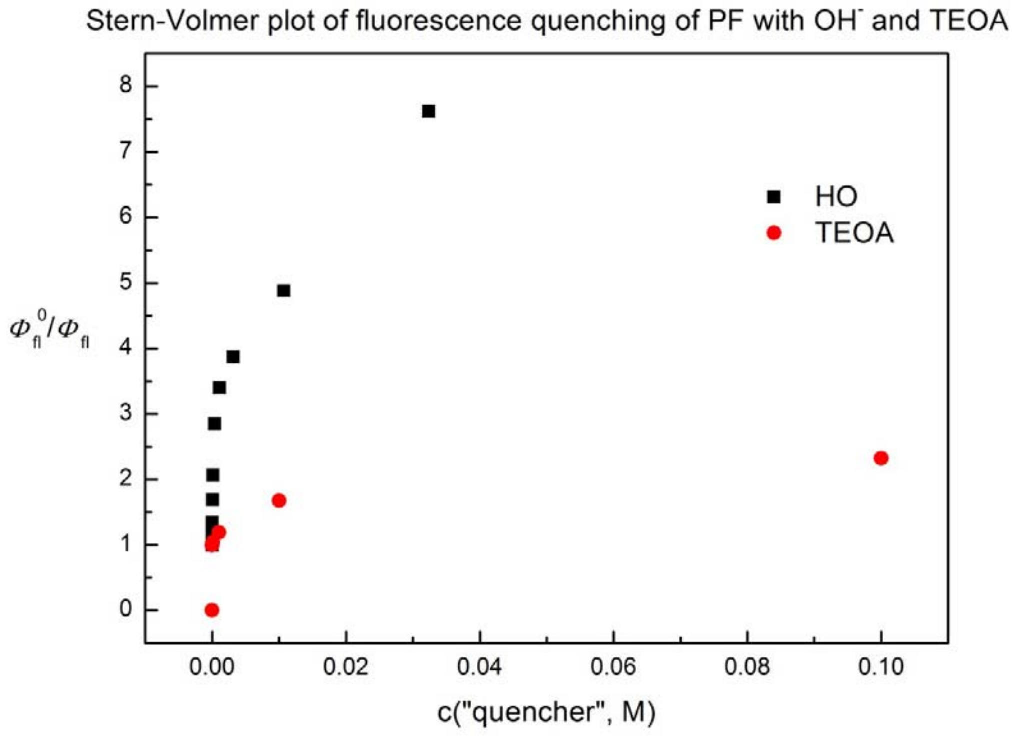


**Figure S21**: Stern-Volmer plot of fluorescence quenching of proflavine hydrochloride with $OH^-$ and TEOA

The dependence of fluorescence intensity on the intensity of excitation light was accomplished by varying the intensity of excitation beam and measuring the intensity of emitted fluorescence. The result is shown in Figure S22. The intensity of excitation light was kept low and all excitation light was absorbed by the sample.

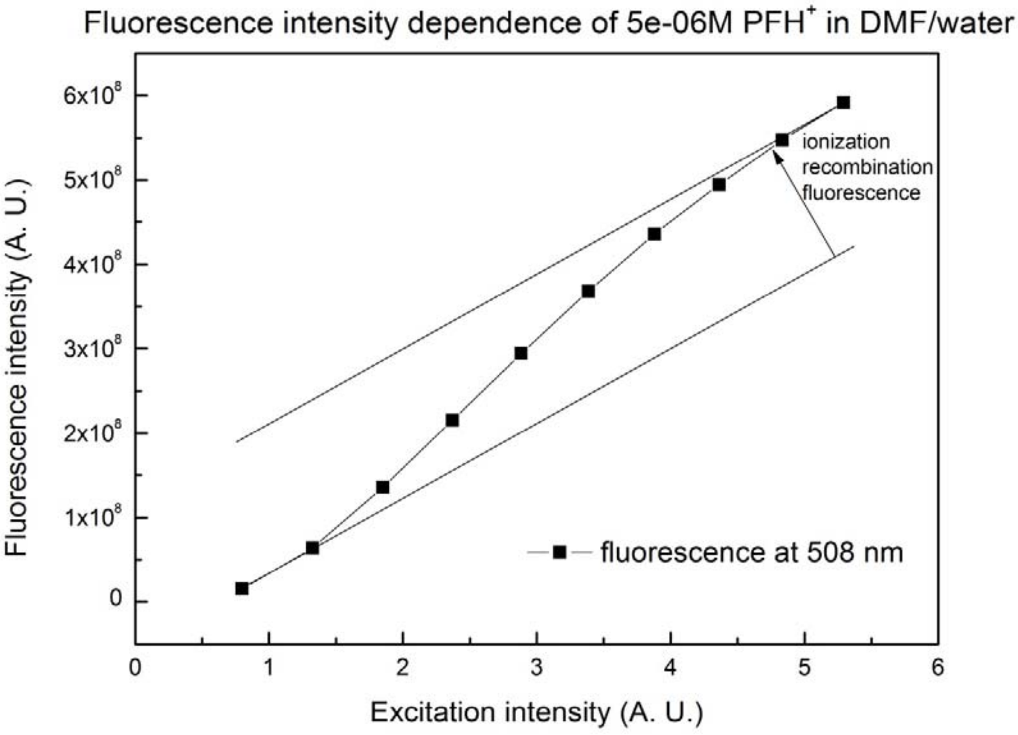


**Figure S22**: Dependence of fluorescence intensity on the intensity of excitation light

The quenching of fluorescence of proflavine hydrochloride with rhodium catalyst is shown in Figure S23.

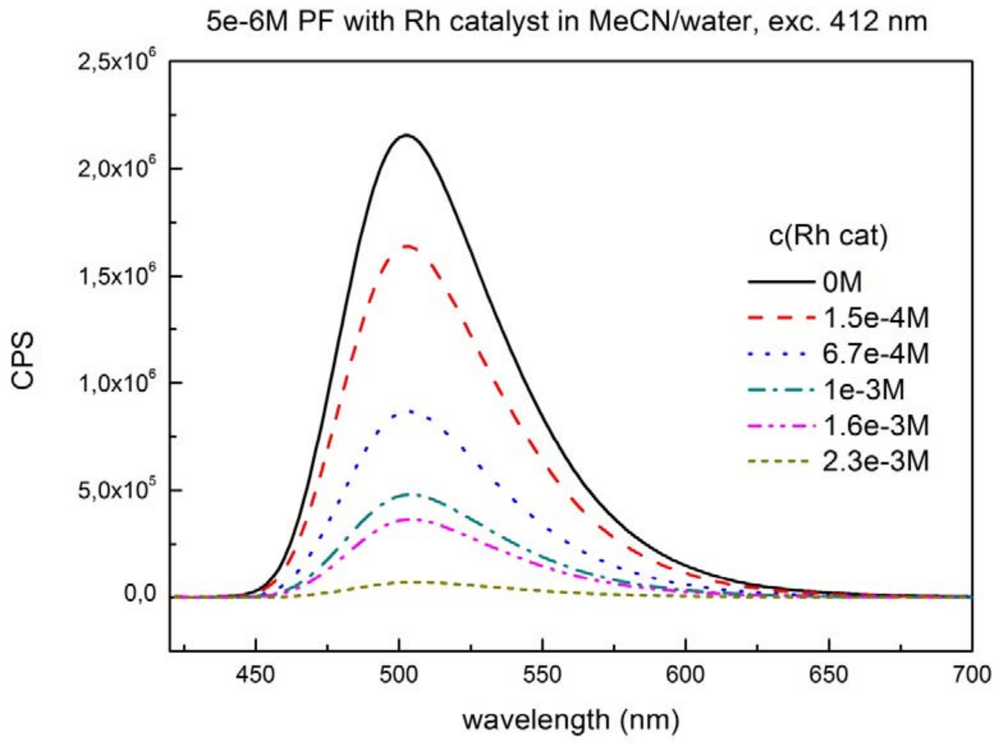


**Figure S23**: Fluorescence quenching of proflavine hydrochloride by rhodium catalyst

The Stern-Volmer plot of the rhodium catalyst quenching is shown in Figure S24. The quenching constant was determined to be ~2200 $M^{-1}$.

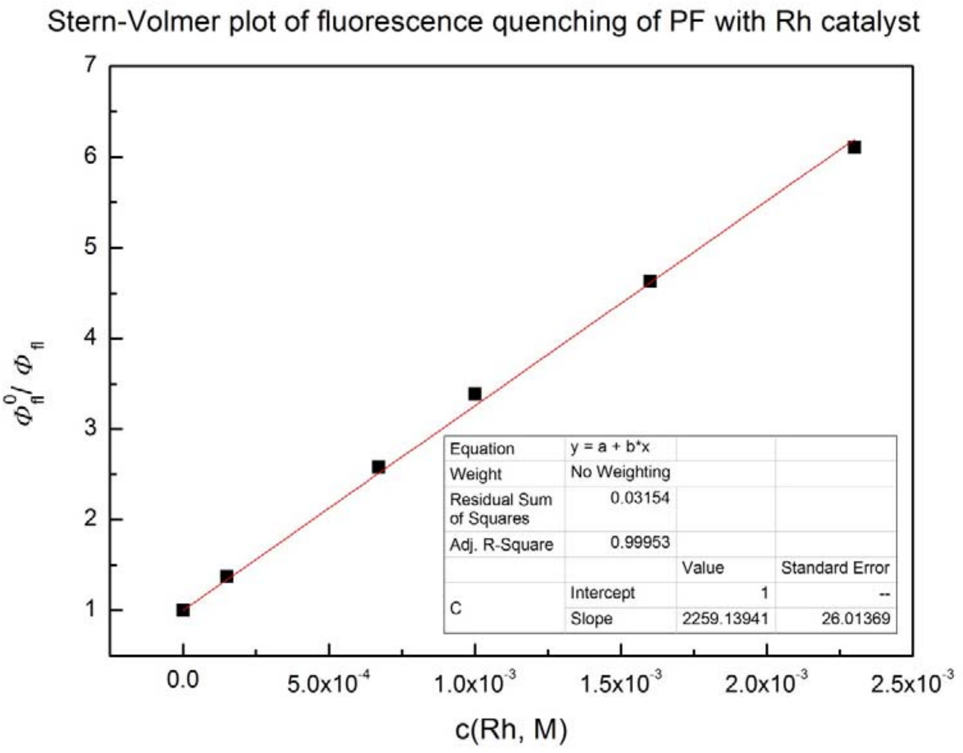


**Figure S24**: Stern-Volmer plot of fluorescence quenching of proflavine hydrochloride with rhodium catalyst

Preparation of RhIII hydride and its characterization

The Rh(III)–H was prepared analogically to the chemical reduction general procedure. No substrate was added to the reaction mixture and the concentration of rhodium catalyst was 0.2 mM.

The UV-Vis spectra of the parent complex and the $Rh^{III}$ hydride are shown in Figure S25.

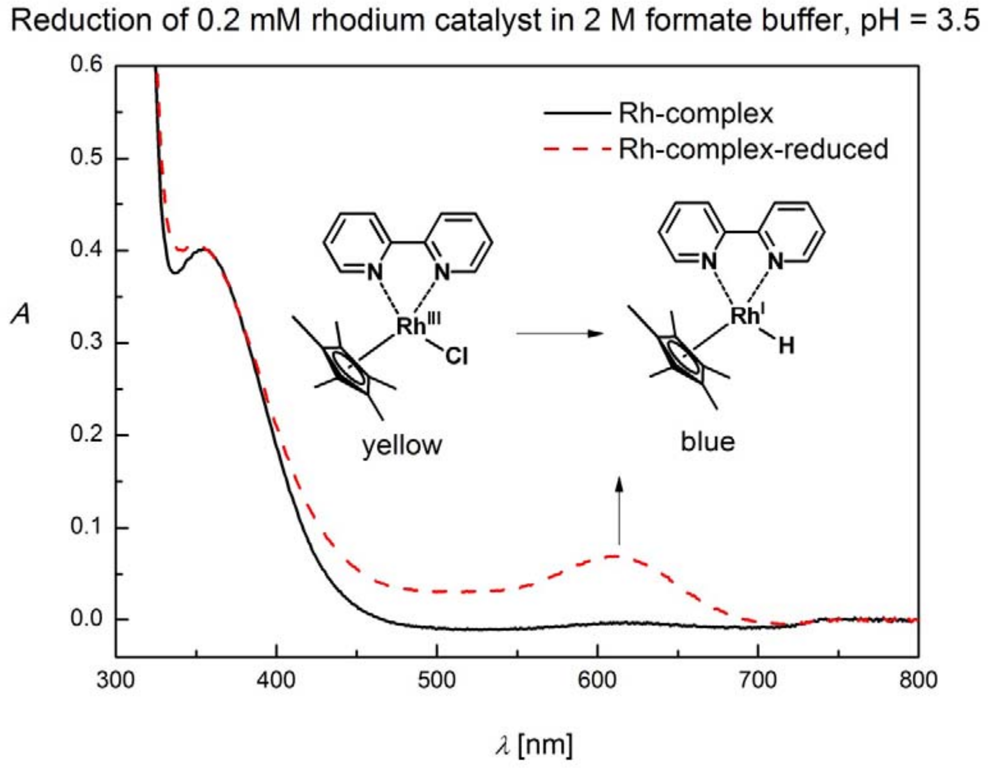


**Figure S25**: UV-Vis spectra of $Rh_{cat}$ and Rh(III)–H

The Rh(III)–H was detected in the typical reaction mixture without substrate after 15 hours of irradiation at 455 nm. After the solution was bubbled with oxygen, the $Rh^{III}$ hydride was re-oxidized and the shoulder at 612 nm disappeared. The results are shown in Figure S26.

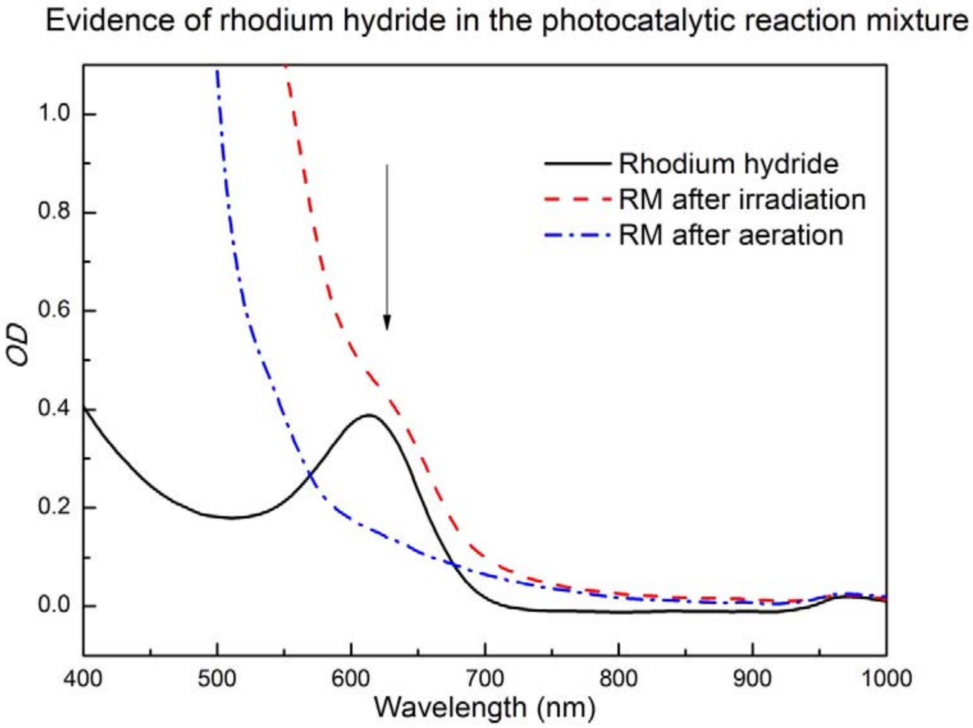


**Figure S26**: UV-Vis evidence of Rh(III)–H present in the photocatalytic reaction mixture after 15 hours of irradiation at 455 nm

Irradiation of proflavine

Proflavine is not photostable. The results of the irradiation of proflavine solutions with various components are shown in Figures S27 – S30. The photoproducts TSG249A1 and TSG249A2 were isolated from a typical reaction mixture without substrate and rhodium catalyst. The photoproducts were not fully characterized; only UV-Vis and fluorescence spectra were measured (Figure S31).

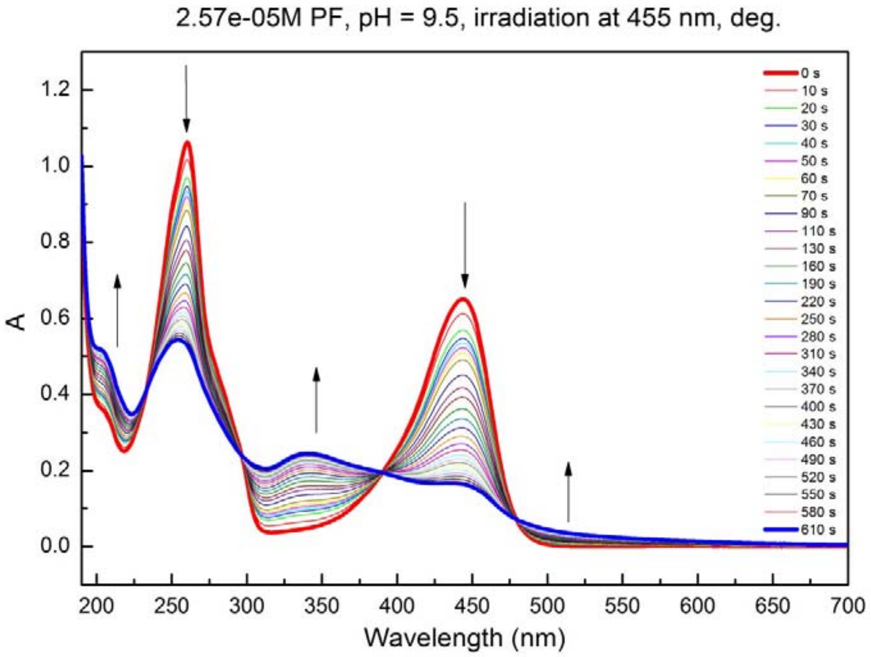


**Figure S27**: UV-Vis spectra of proflavine hydrochloride ($2.57 \times 10^{-5}$ M) irradiated at 455 nm in MeCN/water 1:1

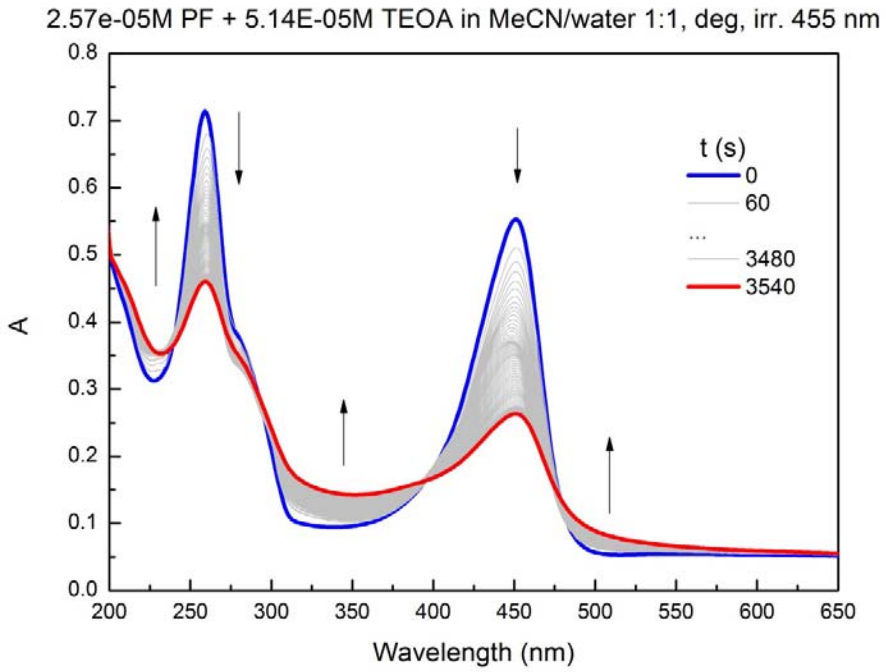


**Figure S28**: UV-Vis spectra of proflavine hydrochloride ($2.57 \times 10^{-5}$ M) and TEOA (2 eq.) irradiated at 455 nm in MeCN/water 1:1

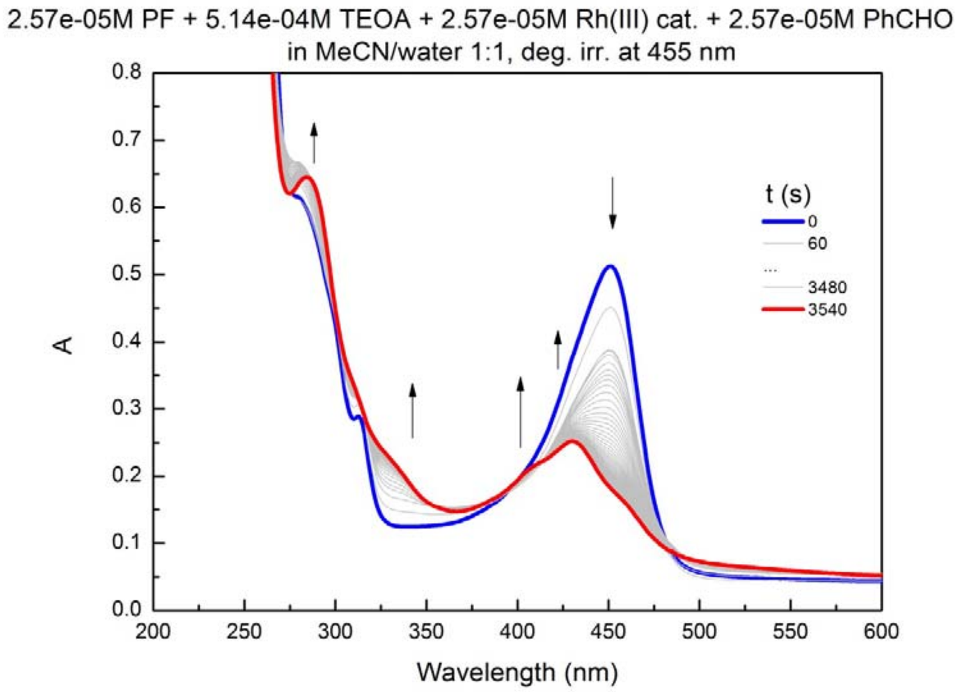


**Figure S29**: UV-Vis spectra of proflavine hydrochloride (2.57 × $10^{-5}$ M), TEOA (2 eq.) and benzaldehyde (2.57 × $10^{-4}$ M) irradiated at 455 nm in MeCN/water 1:1

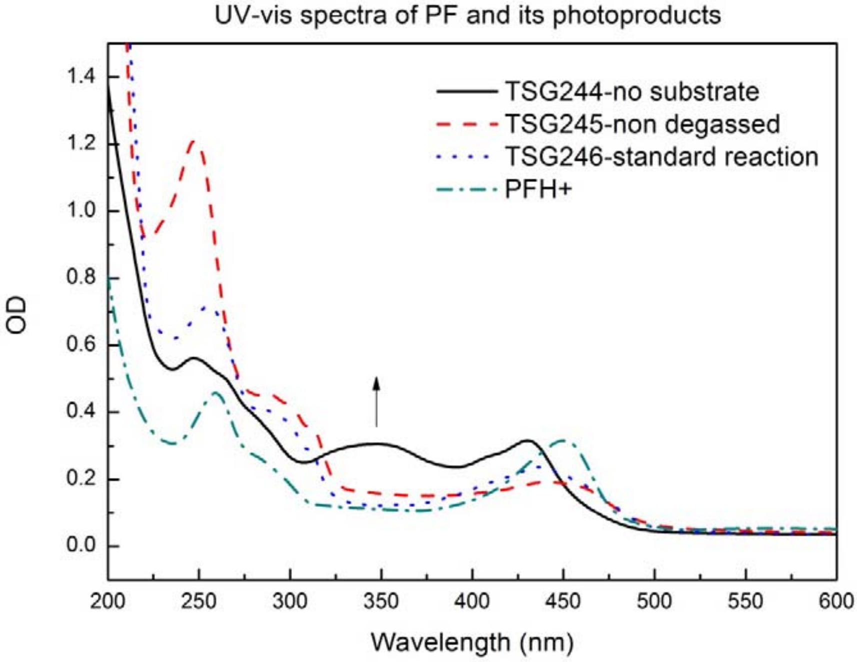


**Figure S30**: UV-Vis spectra of various photoreactions (the typical reaction mixture without substrate and rhodium catalyst– black line; the non-degassed typical reaction mixture – red line; the typical reaction mixture – blue line, proflavine UV-Vis spectrum – green line).

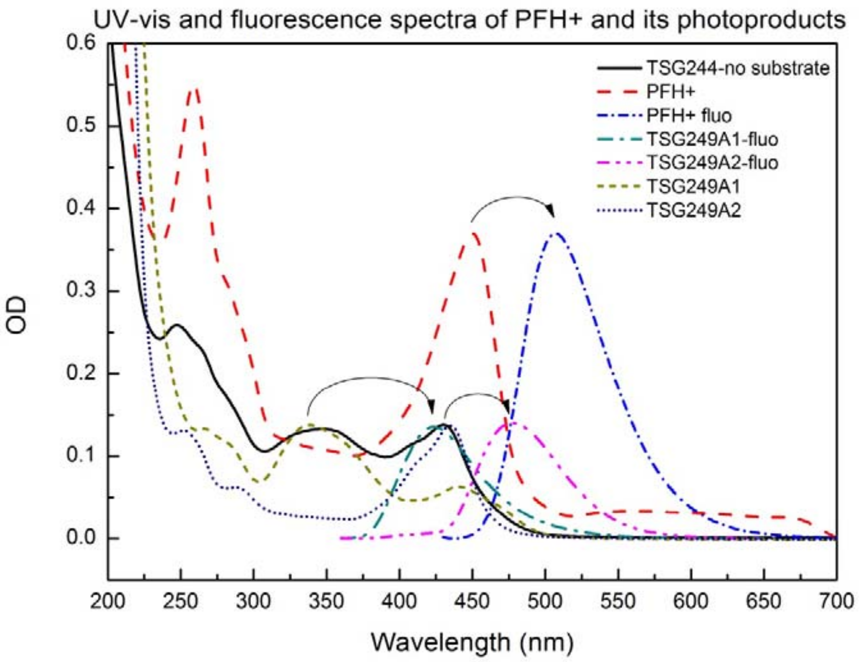


**Figure S31**: UV-Vis spectra of various proflavine (PFH+) photoproducts (TSG249A1 and TSG249A2) and their fluorescence spectra (PFH+ fluo, TSG249A1-fluo and TSG249A2-fluo) normalized to the height of the absorption peak; the curly arrow indicates the Stokes shift. The black solid line corresponds to the photoproduct of the typical reaction without substrate and rhodium catalyst

<u>Transient spectroscopy</u>

The transient spectra were measured with time window of 50 ns (= period of signal accumulation). All spectra were smoothed by Savitzky-Golay method (points of window = 100) in program OriginPro 8.

Transient spectra were measured at various times after the flash. The time is depicted in legend in the figures.

The transient spectra of proflavine triplet are depicted in Figure S32. The triplet was identified according to the characteristic absorption peaks at 550, 610 and 670 nm.[10] The estimated lifetime of proflavine triplet is 2 μs.

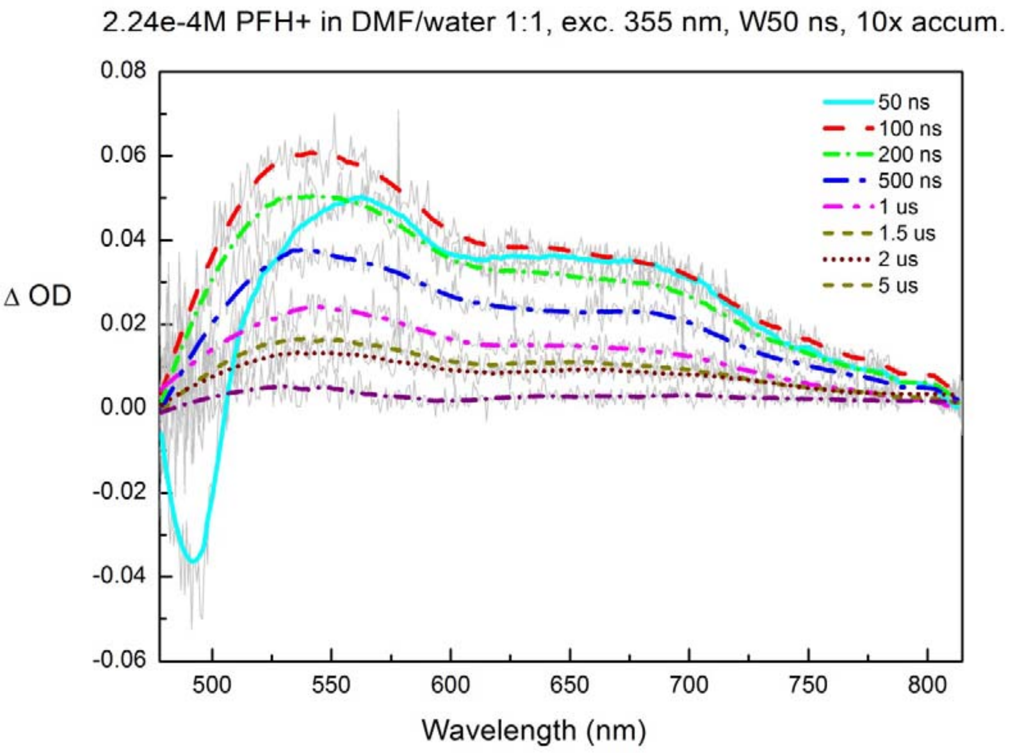


**Figure S32**: Transient absorption spectra of proflavine hydrochloride in DMF/water 1:1, ($c$ = 2.24 × $10^{-4}$ M) bubbled with nitrogen, excitation wavelength $\lambda_{ex}$ = 355 nm; time window 50 ns, 10 × accumulated

The transient spectra of proflavine radical anion are depicted in Figure S33. The radical anion was identified according to the characteristic absorption peak at 530 nm.[9,10] The estimated lifetime of proflavine triplet is 8 μs.

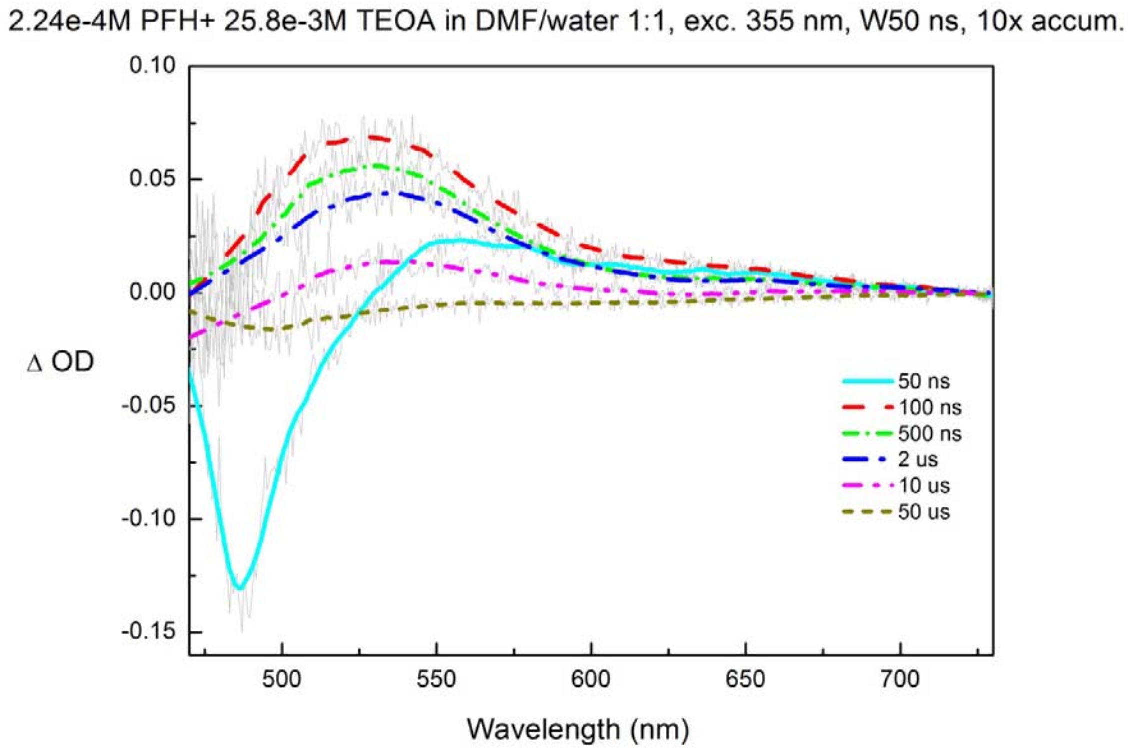


**Figure S33**: Transient absorption spectra of proflavine hydrochloride ($c$ = 2.24 × $10^{-4}$ M) and TEOA ($c$ = 25.8 × $10^{-3}$ M) in DMF/water 1:1, bubbled with nitrogen, excitation wavelength $\lambda_{ex}$ = 355 nm; time window 50 ns, 10 × accumulated

The transient spectra of proflavine hydrochloride with rhodium catalyst are depicted in Figure S34. The triplet is still observed but the intensity is lower.

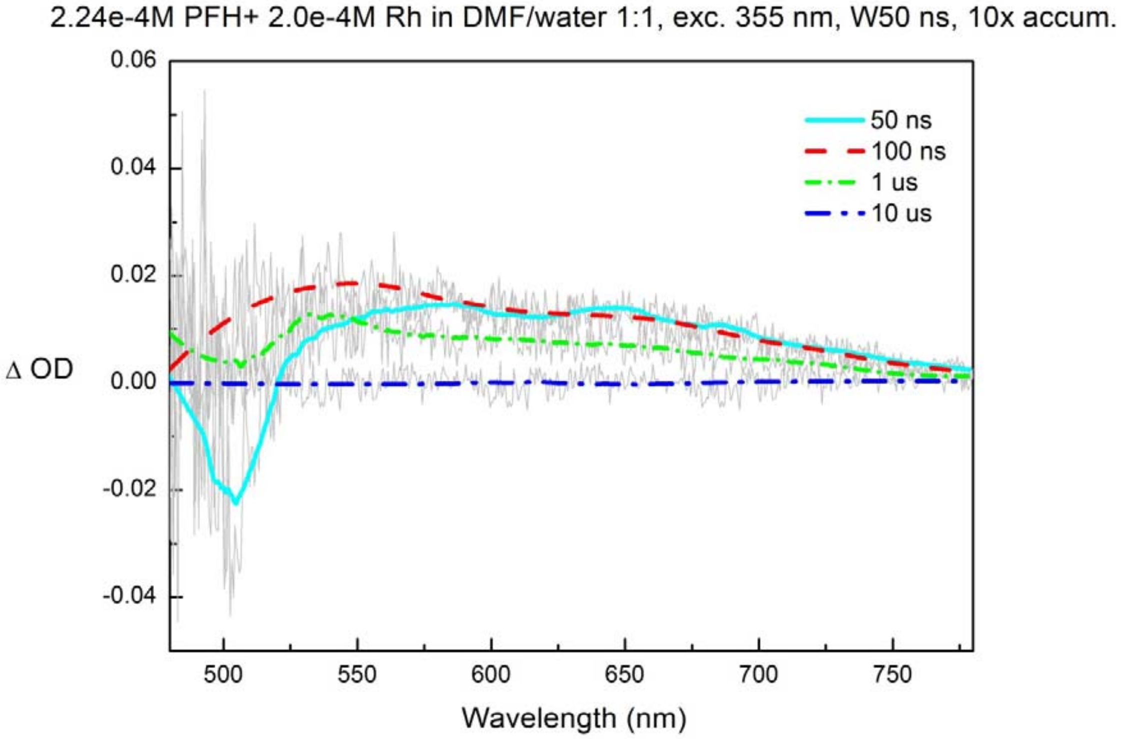


**Figure S34**: Transient absorption spectra of proflavine hydrochloride ($c$ = 2.24 × $10^{-4}$ M) and rhodium catalyst ($c$ = 2.0 × $10^{-4}$ M) in DMF/water 1:1, bubbled with nitrogen, excitation wavelength $\lambda_{ex}$ = 355 nm; time window 50 ns, 10 × accumulated

The transient spectra of proflavine hydrochloride with rhodium catalyst and TEOA are depicted in Figure S35. The proflavine radical anion is observed and the lifetime drops.

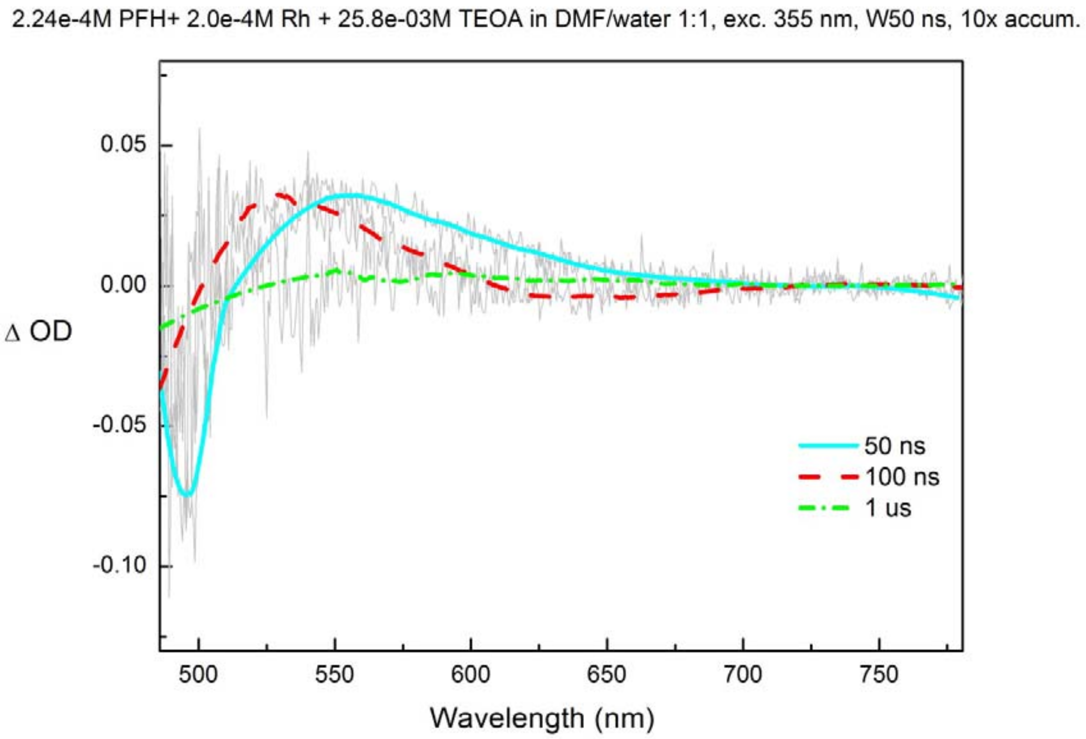


**Figure S35**: Transient absorption spectra of proflavine hydrochloride ($c$ = 2.24 × $10^{-4}$ M), rhodium catalyst ($c$ = 2.0 × $10^{-4}$ M) and TEOA ($c$ = 2.58 × $10^{-2}$ M) in DMF/water 1:1, bubbled with nitrogen, excitation wavelength $\lambda_{ex}$ = 355 nm; time window 50 ns, 10 × accumulated

The transient spectra of rhodium catalyst are depicted in Figure S36.

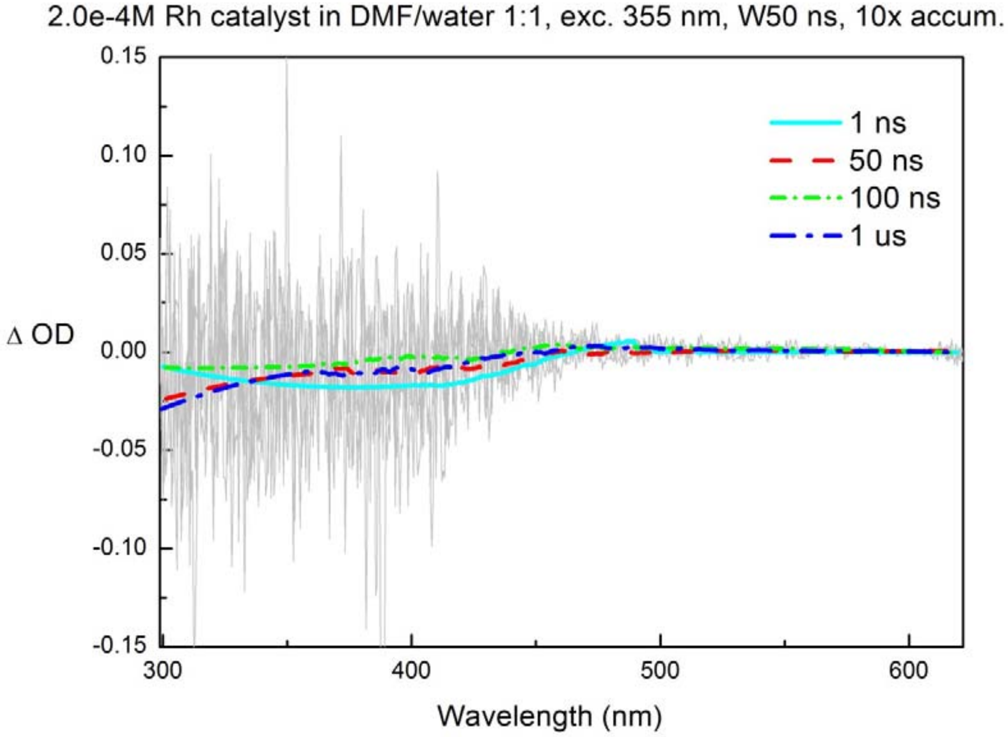


**Figure S36**: Transient absorption spectra rhodium catalyst ($c$ = 2.0 × $10^{-4}$ M) in DMF/water 1:1, bubbled with nitrogen, excitation wavelength $\lambda_{ex}$ = 355 nm; time window 50 ns, 10 × accumulated

The overlay of the signal of the solution of proflavine hydrochloride and the solution of proflavine hydrochloride and rhodium catalyst at 100 ns after the pulse is shown in Figure S37.

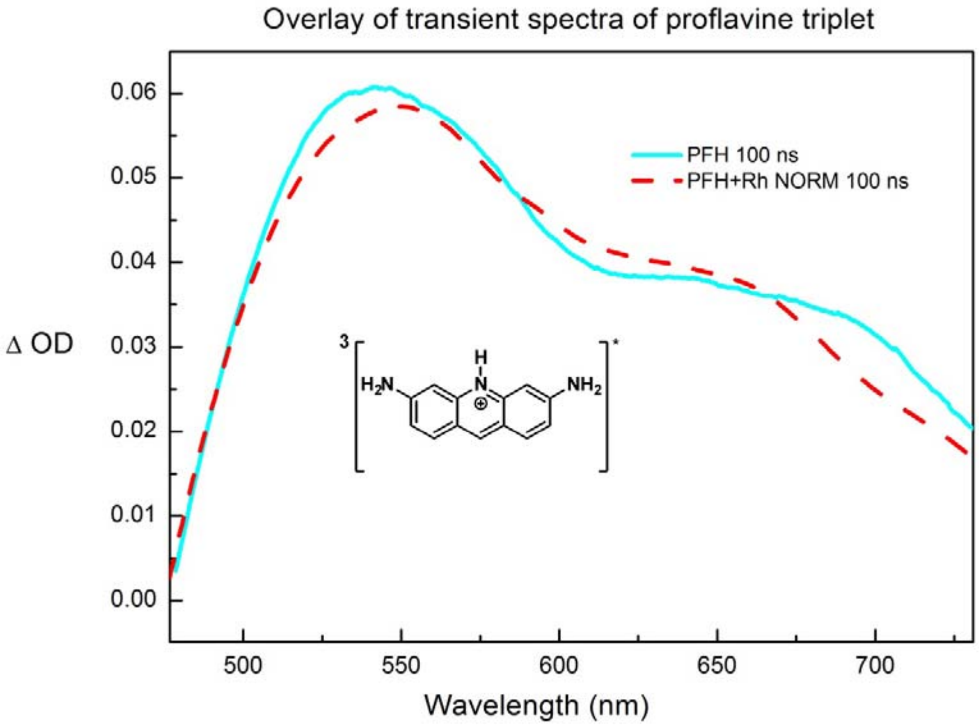


**Figure S37**: Overlay of transient absorption spectra of proflavine hydrochloride and a mixture of proflavine hydrochloride and rhodium catalyst (normalized) at 100 ns after the pulse

The overlay of the signal of the solution of proflavine hydrochloride and TEOA and the solution of proflavine hydrochloride, rhodium catalyst and TEOA (normalized) at 100 ns after the pulse is shown in Figure S38.

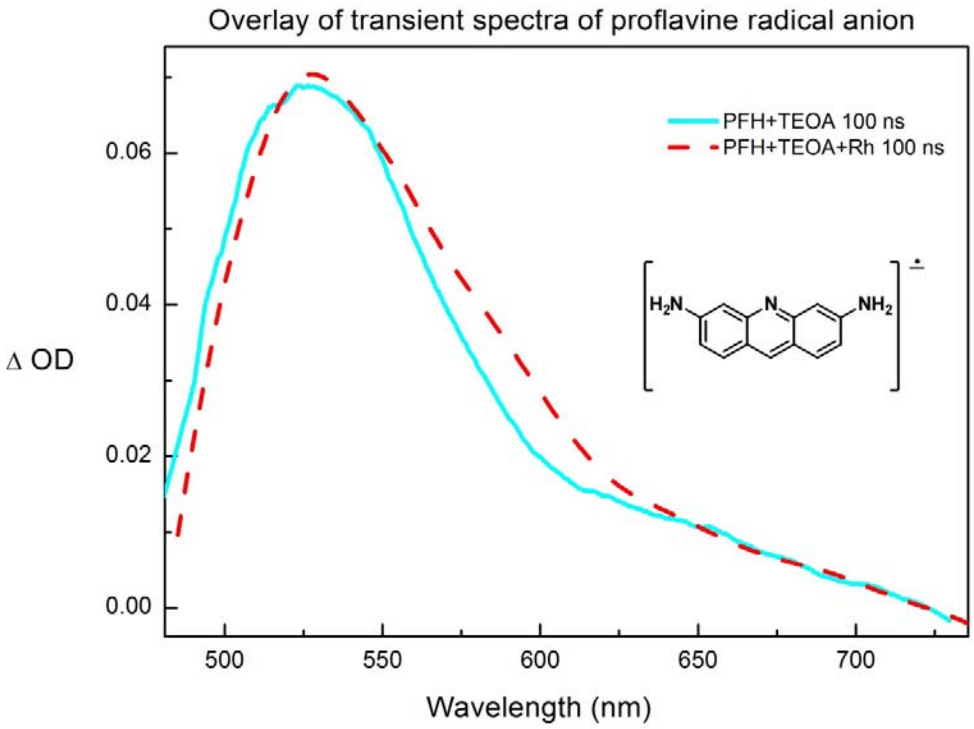


**Figure S38**: Overlay of transient absorption spectra of proflavine hydrochloride and TEOA and the solution of proflavine hydrochloride, rhodium catalyst and TEOA (normalized) at 100 ns after the pulse

References


(1) Megerle, U.; Lechner, R.; Koenig, B.; Riedle, E. *Photochem. Photobiol. Sci.* **2010**, *9*, 1400-1406.

(2) Bonneau, R.; Wirz, J.; Zuberbuhler, A. D. *Pure Appl. Chem.* **1997**, *69*, 979-992.

(3) Ooi, T.; Miura, T.; Itagaki, Y.; Ichikawa, H.; Maruoka, K. *Synthesis* **2002**, *2002*, 279-291.

(4) Chandrasekhar, S.; Shrinidhi, A. *Synth. Commun.* **2014**, *44*, 2051-2056.

(5) Hsu, S.-F.; Plietker, B. *Chem. - Eur. J.* **2014**, *20*, 4242-4245.

(6) Kölle, U.; Grätzel, M. *Angew. Chem., Int. Ed. Engl.* **1987**, *99*, 572-574.

(7) Himeda, Y.; Onozawa-Komatsuzaki, N.; Sugihara, H.; Arakawa, H.; Kasuga, K. *J. Mol. Catal. A: Chem.* **2003**, *195*, 95-100.

(8) Mataga, N.; Kaifu, Y.; Koizumi, M. *Bull. Chem. Soc. Jpn.* **1956**, *29*, 373-379.

(9) Pileni, M. P.; Gratzel, M. *J. Phys. Chem.* **1980**, *84*, 2402-2406.

(10) Kalyanasundaram, K.; Dung, D. *J. Phys. Chem.* **1980**, *84*, 2551-2556.


NMR Spectra and Others

**Figure S39**: $^1$H NMR spectrum of [Cp*Rh(bpy)Cl]Cl

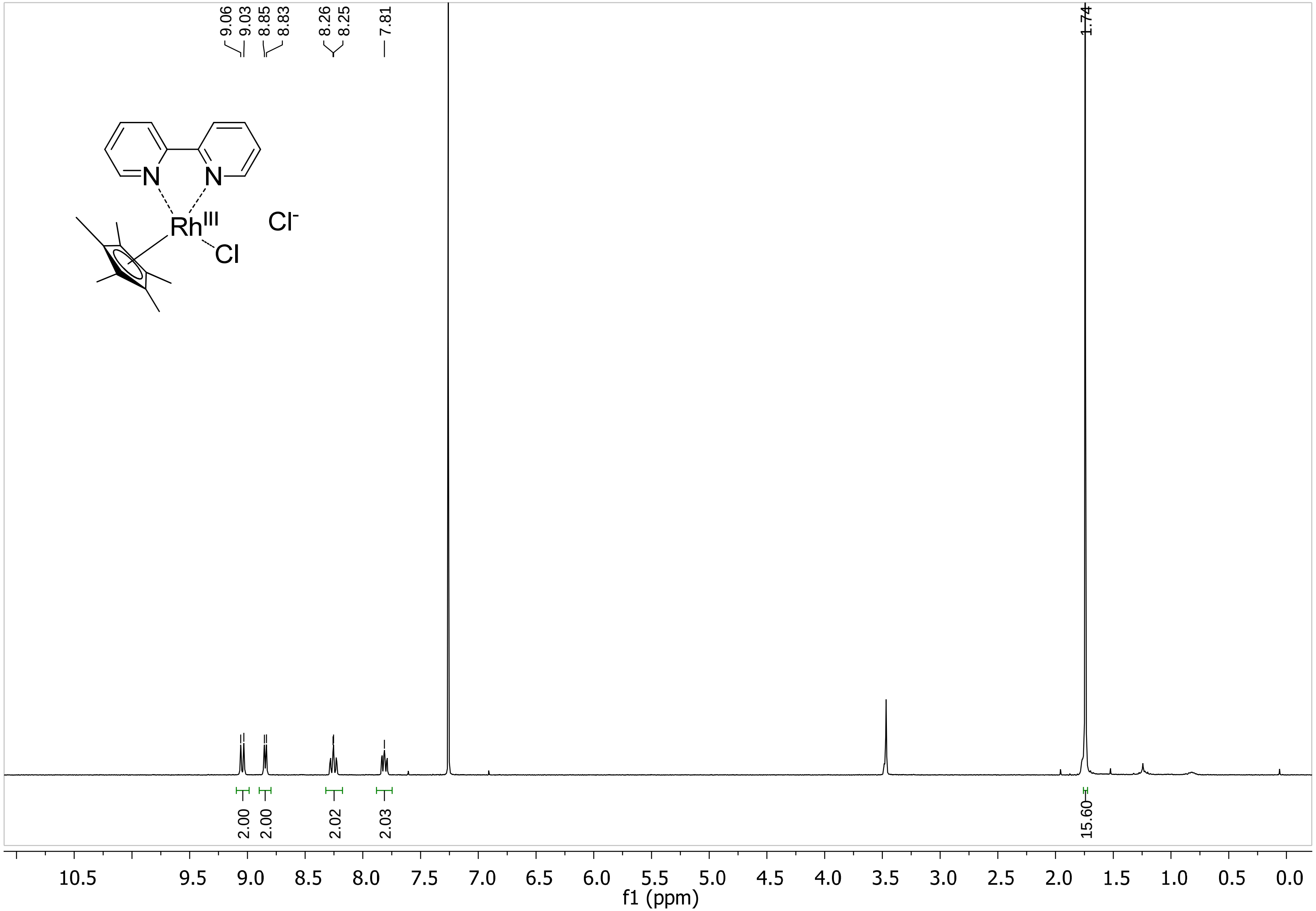

**Figure S40**: $^{1}$H NMR spectrum of 1-(4-(hydroxymethyl)phenyl)ethanone

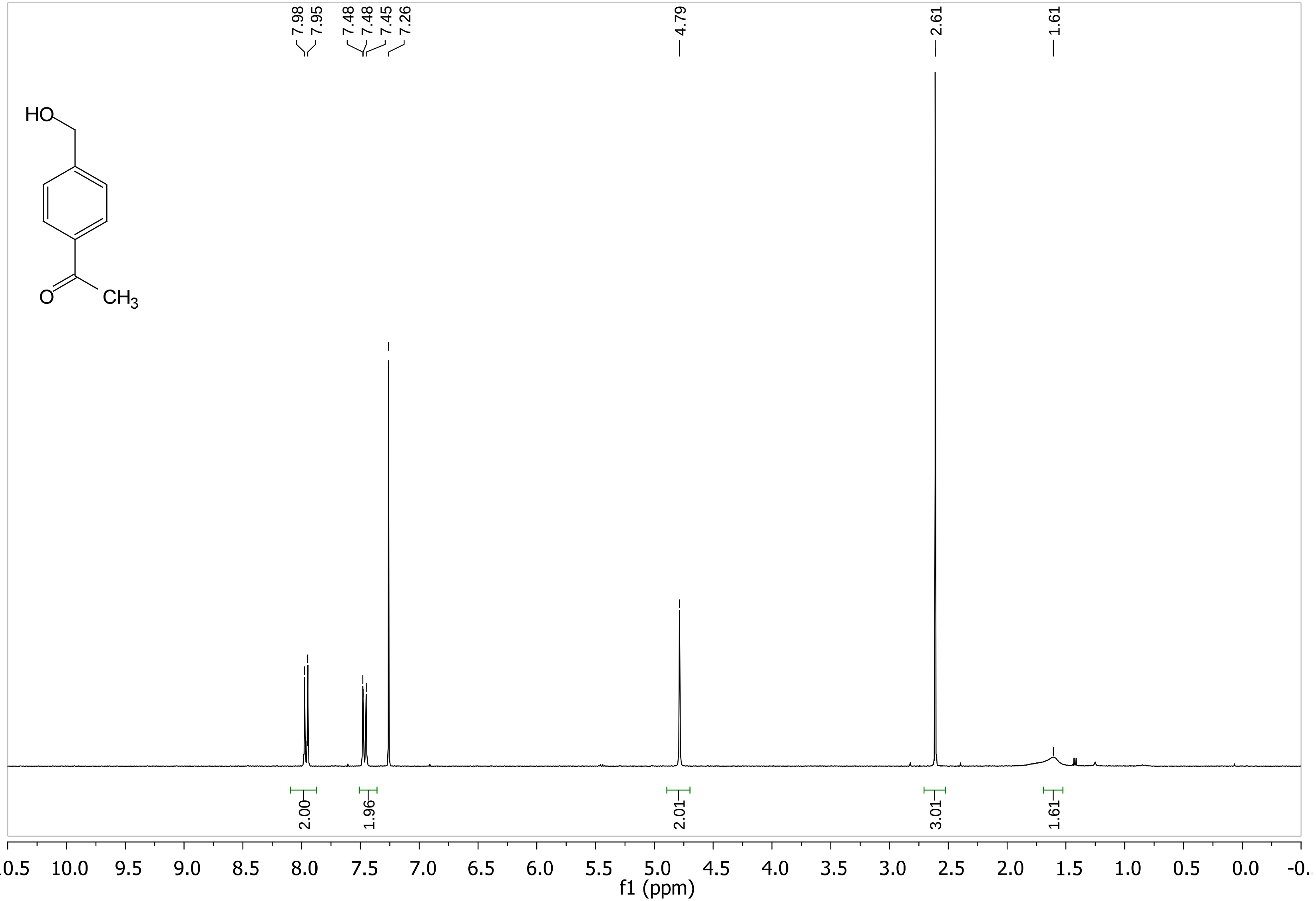

**Figure S41**: $^{13}C$ NMR spectrum of 1-(4-(hydroxymethyl)phenyl)ethanone

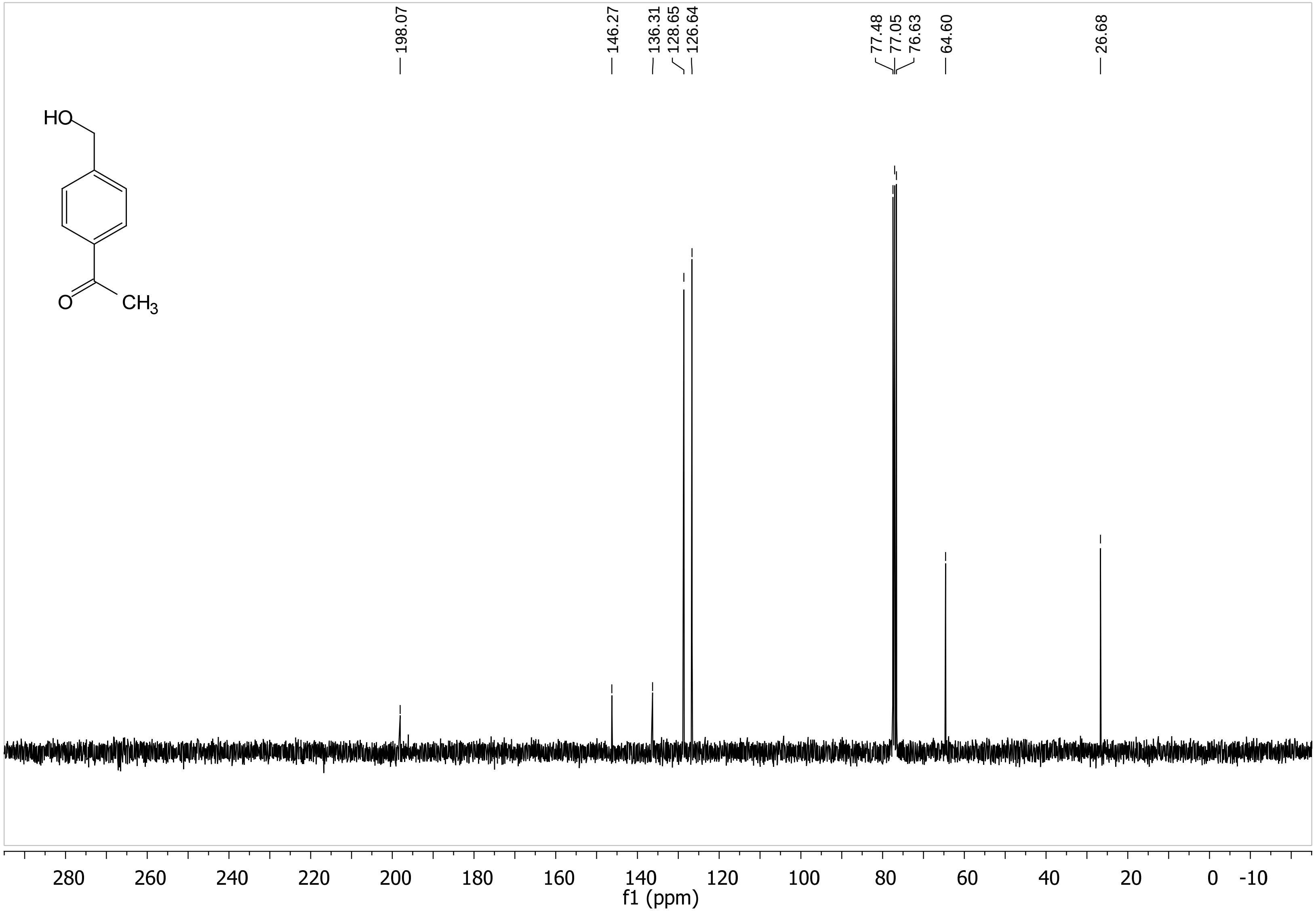

**Figure S42**: $^{1}$H NMR spectrum of methyl 4-(1-hydroxyethyl)benzoate

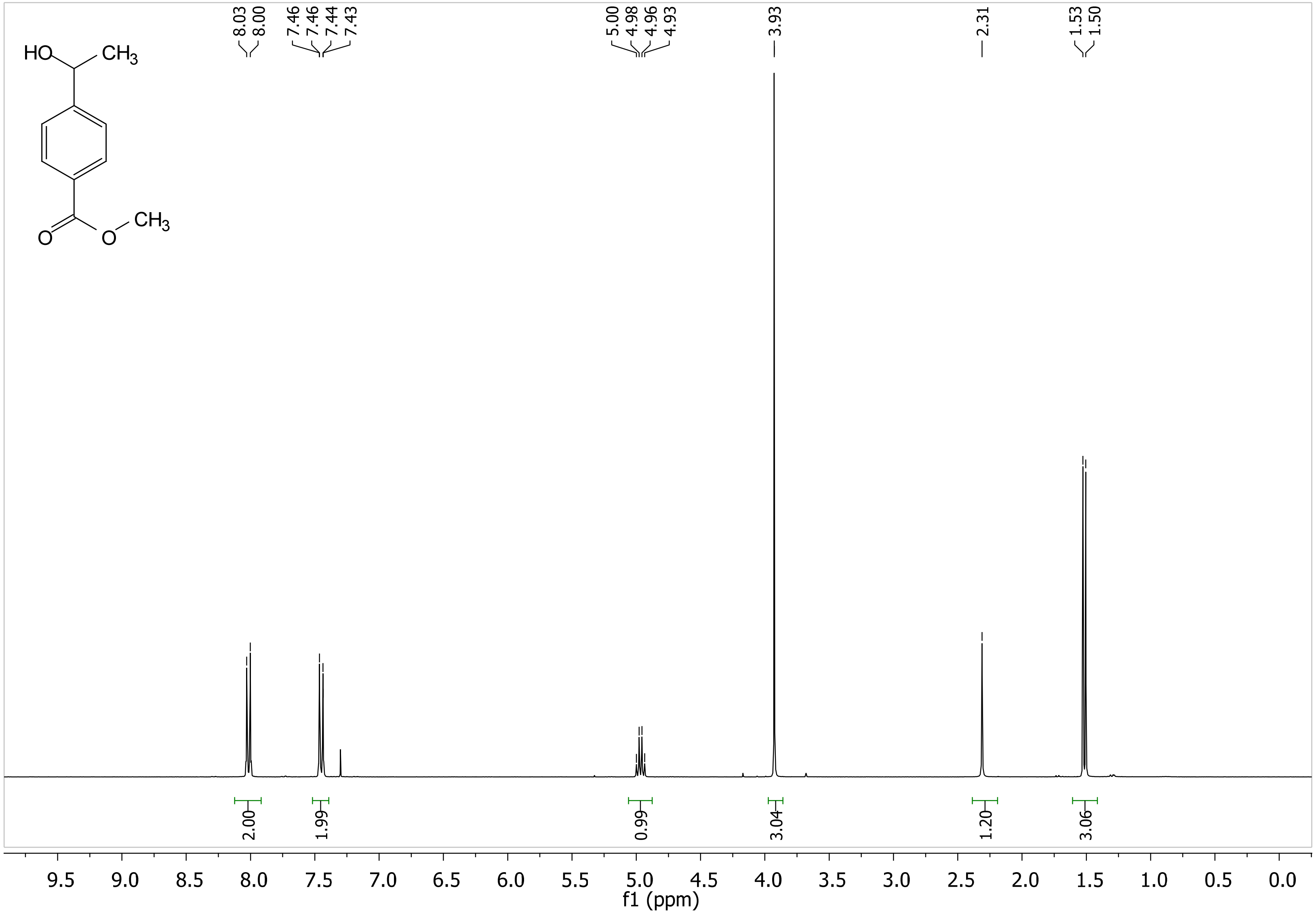

**Figure S43**: $^{13}$C NMR spectrum of methyl 4-(1-hydroxyethyl)benzoate

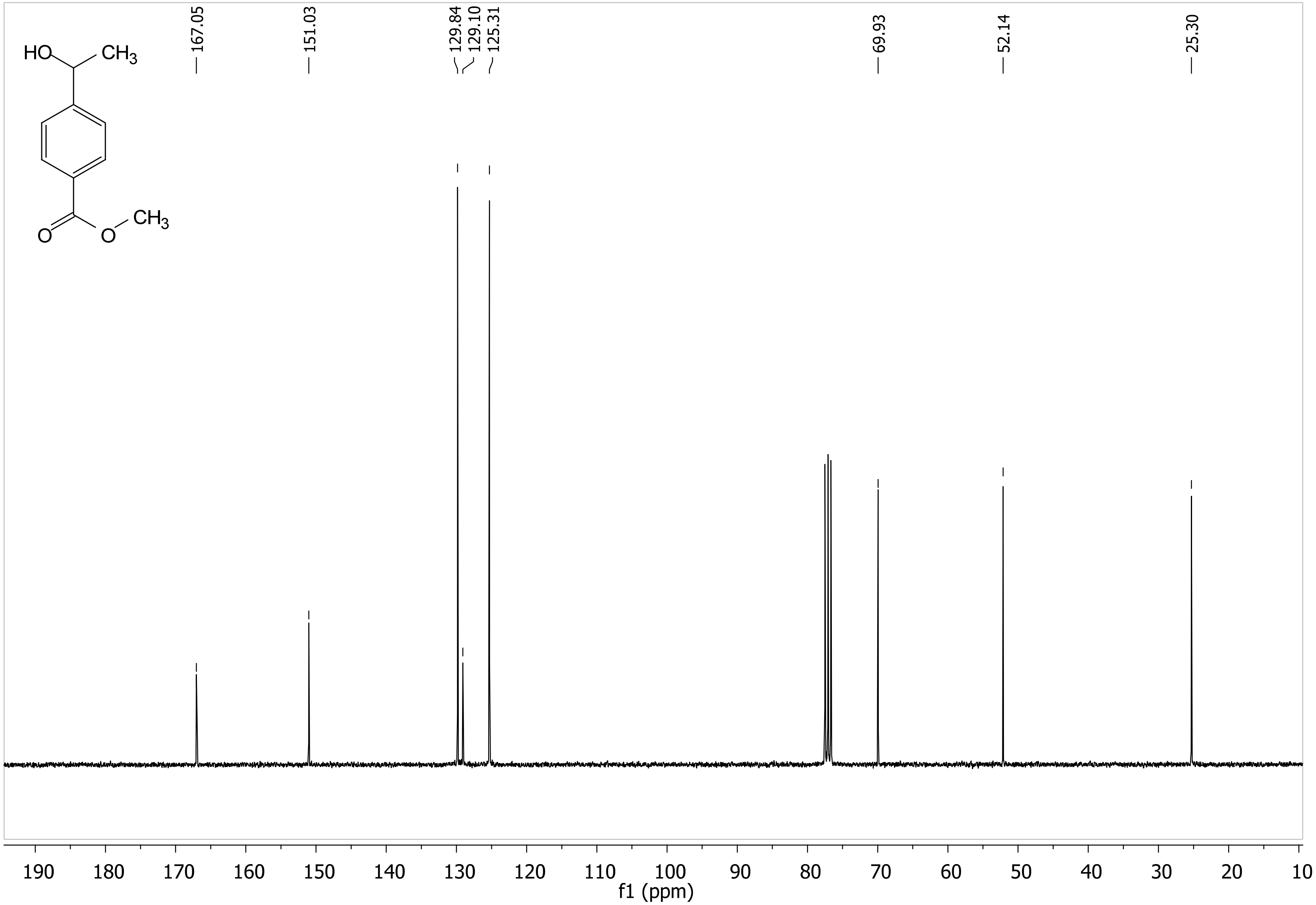

**Figure S44**: HPLC-MS analysis result for the photochemical reduction of 4-fornyl acetophenone; the pinacol products M+H$^+$ (m/z = 299.1; two diastereomers) are indicated

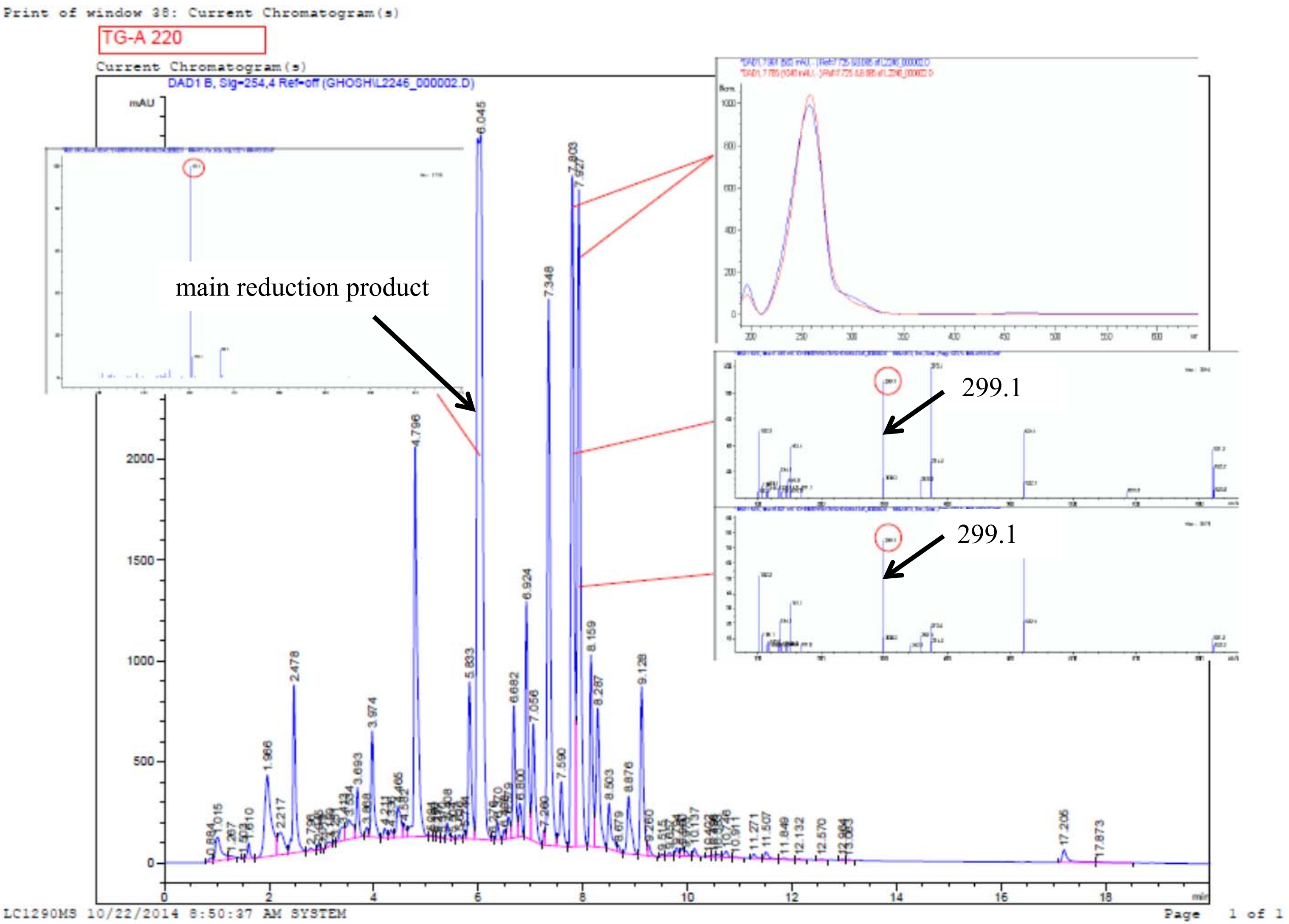

**Figure S45**: Jabłoński diagram of proflavine catalytic system

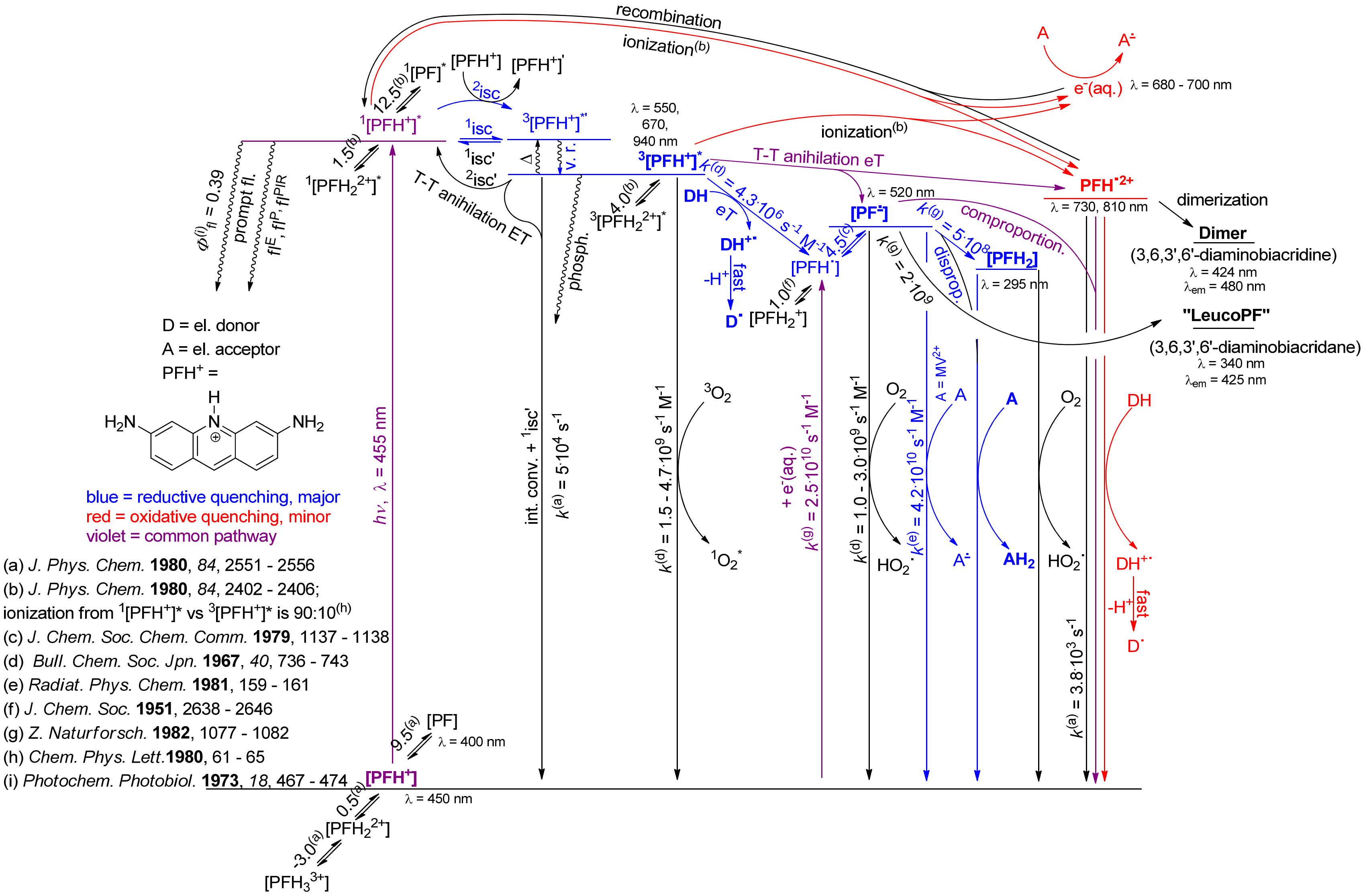

**Figure S46**: Overall mechanism of proflavine catalytic system

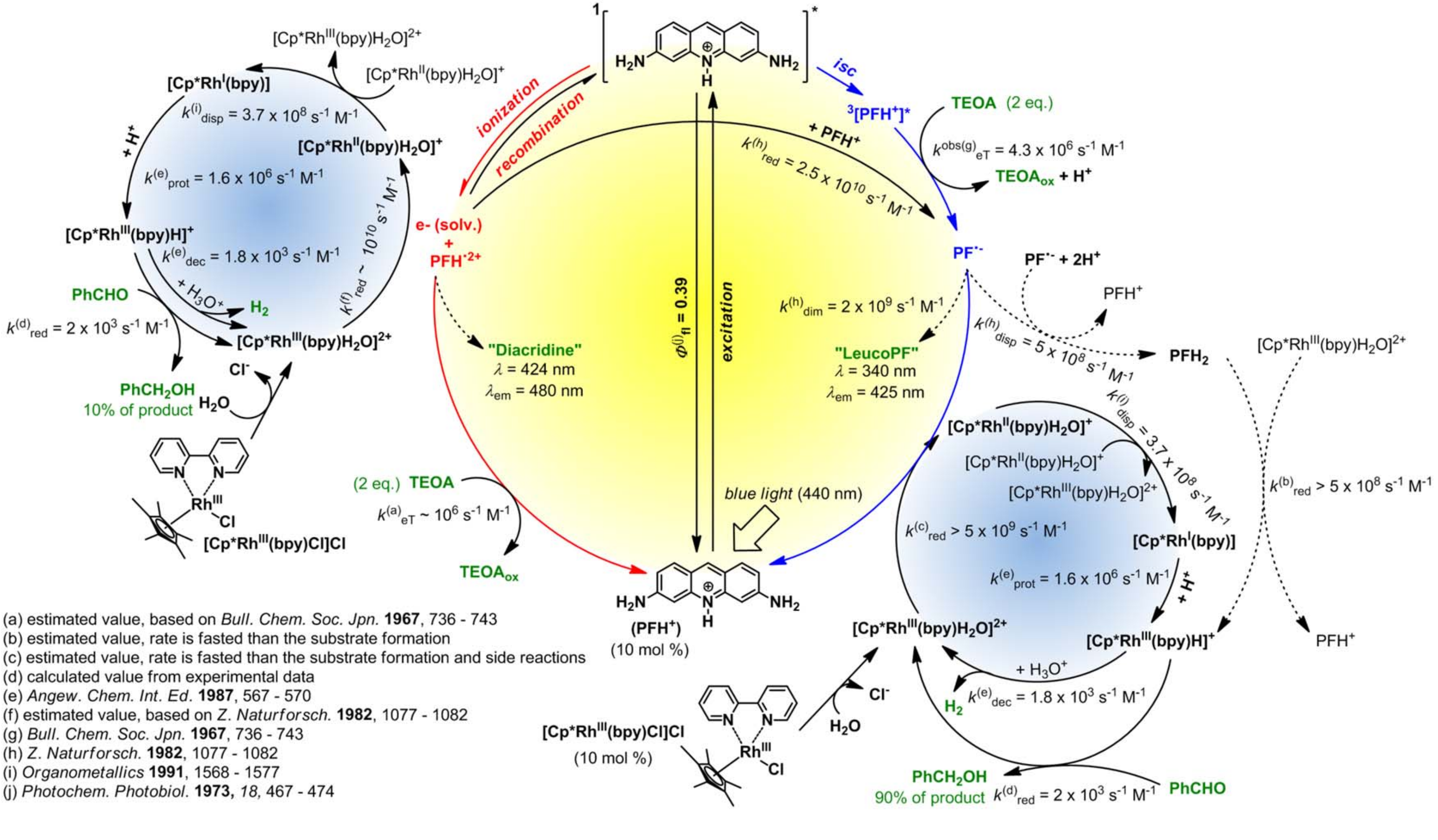


(a) estimated value, based on *Bull. Chem. Soc. Jpn.* **1967**, 736 - 743
(b) estimated value, rate is fasted than the substrate formation
(c) estimated value, rate is fasted than the substrate formation and side reactions
(d) calculated value from experimental data
(e) *Angew. Chem. Int. Ed.* **1987**, 567 - 570
(f) estimated value, based on *Z. Naturforsch.* **1982**, 1077 - 1082
(g) *Bull. Chem. Soc. Jpn.* **1967**, 736 - 743
(h) *Z. Naturforsch.* **1982**, 1077 - 1082
(i) *Organometallics* **1991**, 1568 - 1577
(j) *Photochem. Photobiol.* **1973,** *18,* 467 - 474
